# Metabolism and foraging strategies of mid‐latitude mesozooplankton during cyanobacterial blooms as revealed by fatty acids, amino acids, and their stable carbon isotopes

**DOI:** 10.1002/ece3.5533

**Published:** 2019-08-16

**Authors:** Elvita Eglite, Martin Graeve, Jörg Dutz, Dirk Wodarg, Iris Liskow, Detlef Schulz‐Bull, Natalie Loick‐Wilde

**Affiliations:** ^1^ Department of Biological Oceanography Leibniz‐Institute for Baltic Sea Research Warnemuende Rostock Germany; ^2^ Department of Marine Chemistry Alfred Wegener Institute Helmholtz Centre for Polar and Marine Research Bremerhaven Germany; ^3^ Department of Marine Chemistry Leibniz‐Institute for Baltic Sea Research Warnemuende Rostock Germany

**Keywords:** amino acids, fatty acids, food webs, isoscapes, stable carbon isotopes, zooplankton

## Abstract

Increasing sea surface temperatures (SST) and blooms of lipid‐poor, filamentous cyanobacteria can change mesozooplankton metabolism and foraging strategies in marine systems. Lipid shortage and imbalanced diet may challenge the build‐up of energy pools of lipids and proteins, and access to essential fatty acids (FAs) and amino acids (AAs) by copepods. The impact of cyanobacterial blooms on individual energy pools was assessed for key species temperate *Temora longicornis* and boreal *Pseudo‐/Paracalanus* spp. that dominated field mesozooplankton communities isolated by seasonal stratification in the central Baltic Sea during the hot and the cold summer. We looked at (a) total lipid and protein levels, (b) FA trophic markers and AA composition, and (c) compound‐specific stable carbon isotopes (δ^13^C) in bulk mesozooplankton and in a subset of parameters in particulate organic matter. Despite lipid‐poor cyanobacterial blooms, the key species were largely able to cover both energy pools, yet a tendency of lipid reduction was observed in surface animals. Omni‐ and carnivory feeding modes, FA trophic makers, and δ^13^C patterns in essential compounds emphasized that cyanobacterial FAs and AAs have been incorporated into mesozooplankton mainly via feeding on mixo‐ and heterotrophic (dino‐) flagellates and detrital complexes during summer. Foraging for essential highly unsaturated FAs from (dino‐) flagellates may have caused night migration of *Pseudo‐/Paracalanus* spp. from the deep subhalocline waters into the upper waters. Only in the hot summer (SST>19.0°C) was *T. longicornis* submerged in the colder subthermocline water (~4°C). Thus, the continuous warming trend and simultaneous feeding can eventually lead to competition on the preferred diet by key copepod species below the thermocline in stratified systems. A comparison of δ^13^C patterns of essential AAs in surface mesozooplankton across sub‐basins of low and high cyanobacterial biomasses revealed the potential of δ^13^C‐AA isoscapes for studies of commercial fish feeding trails across the Baltic Sea food webs.

## 1 INTRODUCTION

The recent scenarios of continuous global warming in aquatic ecosystems predict a change in the phytoplankton composition that currently is mostly defined by an increase in cyanobacterial blooms (Huisman et al., 2018; Paerl & Huisman, 2008; Paerl & Paul, 2012). The increase in cyanobacterial blooms might alter the food sources for primary consumers such as mesozooplankton, thus impacting their foraging ecology, nutritional status, and distribution and reproduction of species (Alheit et al., 2005; Edwards & Richardson, 2004; Hansson et al., 2013; Hoegh‐Guldberg & Bruno, 2010; Karjalainen et al., 2007). Most cyanobacteria have commonly been considered to be a lower quality food source for mesozooplankton due to colonies' large size and low levels of lipids compared to other microalgae like diatoms and dinoflagellates (e.g., Ahlgren, Gustafsson, & Boberg, 1992; Finkel et al., 2016; Wannicke, Korth, Liskow, & Voss, 2013). Moreover, the omega‐3 fatty acids (FAs), eicosapentaenoic acid (EPA; 20:5(n‐3)), and docosahexaenoic acid (DHA; 22:6(n‐3)) are two essential and polyunsaturated fatty acids (PUFAs) that enhance zooplankton production (Arendt, Jónasdóttir, Hansen, & Gärtner, 2005; Müller‐Navarra, 2008) but are critically low or even absent in cyanobacteria (Jónasdóttir, 2019). Thus, cyanobacterial blooms impact pelagic food webs in multiple ways. Yet it is largely unclear how mid‐latitude mesozooplankton sustain or build‐up their energy pools like lipids and proteins, and receive PUFAs or essential amino acids (AAs), during unpalatable, lipid‐poor, filamentous cyanobacterial blooms in summer. It can be expected that during summer a sufficient build‐up of lipid storages and PUFAs can be extra challenging. The lipid shortage and imbalanced diet may cause a decrease in the protein levels or change the homeostatic AA composition, a sign of poor quality food and even starvation (Båmstedt & Holt, 1978; Guisande, Maneiro, & Riveiro, 1999; Helland, Christian Nejstgaard, Jørgen Fyhn, Egge, & Båmstedt, 2003; Mayzaud, 1976).

So far the impact of N_2_ fixing, filamentous cyanobacteria on individual energy pools and foraging strategies of mesozooplankton has been assessed mainly through experimental studies (Brett & Müller‐Navarra, 1997; Koski, Engström, & Viitasalo, 1999; Loick‐Wilde et al., 2012; Wannicke et al., 2013) and fewer field studies (Eglite et al., 2018; Hogfors et al., 2014; Loick‐Wilde et al., 2019). None of these studies have looked simultaneously on the impact of cyanobacteria on mesozooplankton with different energy strategies. The central Baltic Sea can be taken as a natural model to explore the consequences of persistent cyanobacteria blooms on different energy metabolisms of mid‐latitude mesozooplankton (Conley, 2012; Karlson et al., 2015; Reusch et al., 2018). Here, euryhaline, temperate *Temora longicornis*, and boreal *Pseudocalanus* spp. (also *Paracalanus* spp. that often included in *Pseudocalanus* group, Schulz et al., 2012) are usually dominant calanoid copepods in bulk mesozooplankton composition (Corkett & McLaren, 1979; Frost, 1989; Hernroth & Ackefors, 1979). Both copepod species differ in their principal source of energy storages that are used during reproduction or overwintering periods with low food availability (Peters, Dutz, & Hagen, 2013; Peters, Renz, Van Beusekom, Boersma, & Hagen, 2006). *T. longicornis* typically has a high‐protein content (Evjemo, Reitan, & Olsen, 2003) and accumulates lipids mainly as triacylglycerol (Kattner, Krause, & Trahms, 1981; Peters et al., 2013), while *Pseudocalanus* spp. accumulates large amounts of wax ester (Peters et al., 2006), but their protein status is currently unknown. In the Baltic Sea, both lipid‐accumulating copepod species are essential in the diet for commercial fishes like herring and sprat (Bernreuther et al., 2018).

Investigations into lipid and protein pools in mesozooplankton can help us to disentangle dietary preferences, foraging strategies, and trophic networks. Relative quantities of FA trophic markers can help to distinguish between FAs that are incorporated by mesozooplankton from the most common phytoplankton groups such as diatoms, dinoflagellates, and cyanobacteria (Dalsgaard, St. John, Kattner, Müller‐Navarra, & Hagen, 2003). The FAs can also indicate an input of detrital matter in the animals' diet (Kattner & Krause, 1989). However, FA trophic markers are not taxonomic indicators at the phytoplankton species level because some FAs indicate multiple algae classes (e.g., EPA in diatoms and (dino‐) flagellates, Dalsgaard et al.., 2003). Further, certain FAs like 18:1(n‐9), that are typically found in *Pseudocalanus* spp. in the Baltic Sea, can still be synthesized de novo by zooplankton (Kattner & Hagen, 1998; Peters et al., 2006). Lately, stable carbon isotope analysis (δ^13^C) of individual essential compounds has been used as an accurate tool to reconstruct phototrophic sources of FAs and AAs in food webs (Kohlbach et al., 2016; Larsen et al., 2013; McMahon, Fogel, Elsdon, & Thorrold, 2010; Nielsen, Clare, Hayden, Brett, & Kratina, 2018) and thus can complement studies on FA trophic markers. Typically, particulate organic matter (POM) comprises a mixture of dietary sources, for example, including FAs and AAs from bacteria and algae taxa (Larsen et al., 2013). The combination of δ^13^C values of FAs together with information of diatom‐, (dino‐) flagellates‐, and cyanobacteria FA trophic markers can help to distinguish the major dietary FA source for zooplankton (Budge et al., 2008; Kohlbach et al., 2016), while the unique δ^13^C patterns of essential AAs of phylogenetically distinct groups, like bacteria and microalgae, can be used as end‐members to reveal the primary AA sources for mesozooplankton from fresh algae or microbially reworked POM (Larsen, Taylor, Leigh, & O'Brien, 2009; Larsen et al., 2013). Moreover, the δ^13^C patterns of essential AAs in zooplankton can be used to define regionally specific planktonic food webs for geographic “isoscapes” (Graham, Koch, Newsome, McMahon, & Aurioles, 2010; Hobson, Barnett‐Johnson, & Cerling, 2010; McMahon & Newsome, 2019; Nielsen et al., 2018; Vokhshoori, Larsen, & McCarthy, 2014). Particularly in the Baltic Sea, it is expected that δ^13^C patterns of essential AAs in mesozooplankton might be different between more saline or brackish areas due to associated blooms of diatoms and (dino‐) flagellates versus cyanobacteria from the southwest to the central Baltic Sea, respectively (Loick‐Wilde et al., 2019; Wasmund, Dutz, Pollehne, Siegel, & Zettler, 2015, 2016).

The goal of this study was to identify in field conditions how mesozooplankton with different energy metabolism cover their lipid and protein demands at times of unpalatable, lipid‐poor, filamentous cyanobacteria bloom in mid‐summer. We focused on two Baltic key species *T. longicornis* and *Pseudo‐/Paracalanus* spp. that largely dominated the mesozooplankton community during two contrasting summers (in the hot summer and the cold summer of 2014 and 2015, respectively) at the Eastern Gotland Basin (EGB) of the central Baltic Sea. Based on vertical taxonomic and biomass data, we identified potentially feeding habitats in the water column. We compared lipid and protein levels between both summers and with earlier studies to discuss whether the energy pools are sufficient or exhausted during lipid‐poor cyanobacterial blooms. In order to identify the main food sources of *T. longicornis* and *Pseudo‐/Paracalanus* spp.‐dominated mesozooplankton communities during a period of cyanobacterial bloom, we characterized the phytoplankton‐specific FA trophic markers (Peters et al., 2013, 2006). We complemented the information of dietary sources by the patterns of δ^13^C of essential FAs and AAs in mesozooplankton, and in a subset of parameters in POM. In this study for the first time, we also compared the δ^13^C patterns of essential AAs in mesozooplankton from the EGB with additional δ^13^C‐AA data in mesozooplankton from other sub‐basins and discussed the potential use of δ^13^C‐AA isoscapes in the Baltic Sea.

## 2 MATERIALS AND METHODS

### 2.1 Study area and sampling

**Figure 1:**
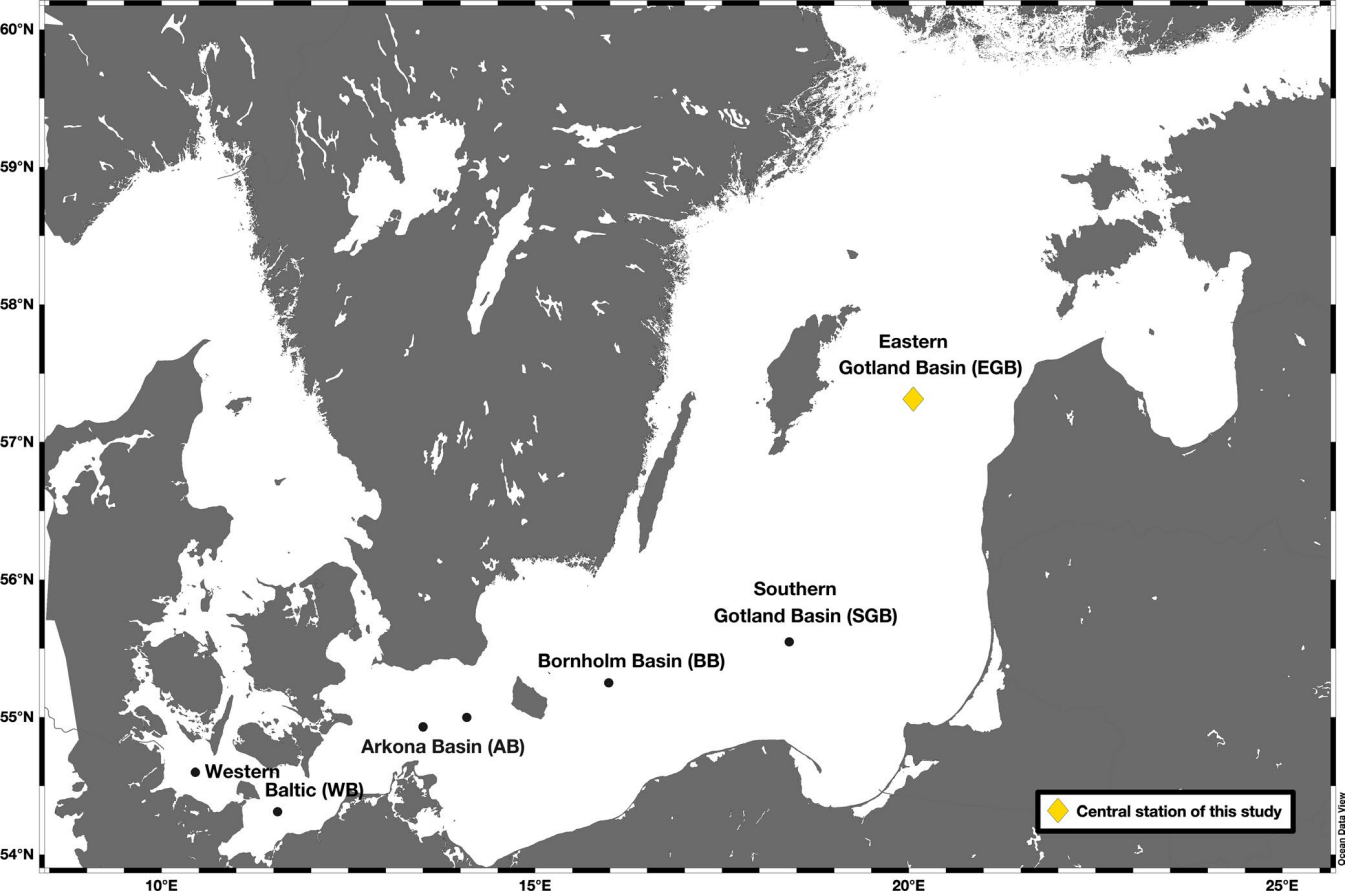
Sampled stations across the five sub‐basins of the Baltic Sea during two cruises on board of R/V *Elisabeth Mann Borgese* on July 24–25 in 2014 and on board of R/V *Meteor* on July 30–31 in 2015. The core study site was the Eastern Gotland Basin (EGB, yellow diamond). In the Western Baltic (WB) and the Arkona, Bornholm, and Southern Gotland Basins (AB, BB, SGB), samples were taken only for basin‐specific “isoscapes” based on the δ^13^C values of essential AAs in mesozooplankton (black dots)

Mesozooplankton and POM samples were collected at mid‐latitudes in the Eastern Gotland Basin (EGB) of the central Baltic Sea (57°N19′N, 20°03′E; Figure 1) during two summer cruises on board of R/V *Elisabeth Mann Borgese* on July 24 and 25 in 2014 and on board of R/V *Meteor* on July 30 and 31 in 2015 (Figure 1). Not all biochemical analyses were applied for all samples. For POM, stable C isotope values (expressed as δ^13^C) in bulk and individual AAs were analyzed. Mesozooplankton samples were analyzed for taxonomy and biomass, total lipid and protein content, composition of individual FAs (including trophic markers) and AAs, content of fatty alcohols (FAlc), content of wax ester lipid class, and for δ^13^C values in bulk and individual FAs and AAs. Further, we tested whether we can identify basin‐specific “isoscapes” across the Baltic Sea based on the δ^13^C patterns of essential AAs in mesozooplankton that were additionally collected in the western (Kiel and Mecklenburg Bights, summarized as Western Baltic: WB) and eastern parts (Arkona, Bornholm, and Southern Gotland Basins: AB, BB, SGB) of the Baltic Sea in 2015 (Figure 1).

The hydrographic properties were measured using a Seabird SBE‐911+CTD unit (Seabird Electronics) equipped with sensors for conductivity, temperature, pressure, and oxygen. In the EGB, we sampled three water bodies that are typically formed in the water column during the summer stratification and which can be easily identified from the temperature, salinity, and oxygen profiles. Sampling depth intervals for POM and mesozooplankton were adjusted to hydrography covering the water column from the thermocline to the surface (surface water: SW, at stations across all sub‐basins), from the halo‐ to the thermocline, that comprises the intermediate cold water that developed during the previous wintertime (“winter water”: WW, at the EGB only; Leppäranta & Myrberg, 2009) and from the oxy‐ to the halocline (bottom water: BW, at the EGB only). At the additional sub‐basin stations (WB, AB, BB, SGB), mesozooplankton was collected from the mixed surface water layer during daytime only.

In the EGB, samples of POM were taken from the Chl. a maxima in the SW and additional subsurface depths (WW) during both years by filtrating 0.5–1 L of seawater through precombusted Whatman GF/F filters (0.7 μm pore size, 25 mm in diameter) and 5–10 L of seawater through polycarbonate Nuclepore filters (0.8 μm pore size, 47 mm in diameter) for elemental (total carbon and total nitrogen) and biochemical (AAs) analyses, respectively. All filters were stored at −20°C after shock‐freezing in liquid nitrogen (−196°C). Unfortunately, POM samples did not contain enough material for additional FA analyses.

Mesozooplankton was collected using a WP‐2 net (mesh size 100 µm, 0.26 m^−2^ opening, vertically towed at 0.2 m/s) during the day and at night (EGB only) according to the Helcom COMBINE Programme (HELCOM, 2017). In the EGB, mesozooplankton from the BW was sampled for biochemical analyses in 2014 only. Later in the text, the mesozooplankton communities from the SW and the WW together are termed “upper water community (UWC).”

Sampling for mesozooplankton taxonomy and subsequent taxon‐specific wet weight estimation was part of the Baltic Sea monitoring program, and the analysis was done according to the methodology guidelines of the Helsinki Commission's (HELCOM) manual (COMBINE) for the protection of the marine environment of the Baltic Sea (HELCOM, 2017). For mesozooplankton taxonomic analyses in the Eastern Gotland Basin, only the daytime mesozooplankton samples from each depth were stored and preserved in 4% borax‐buffered formaldehyde according to HELCOM (2017). Each sample was analyzed until 100 individuals of the three dominant zooplankton taxa (excluding cladocerans, nauplii, rotifers, and tintinnids) were counted. The precision of abundance when counted to 100 individuals of a taxon is 20% (see table 3 in HELCOM, 2017). The estimation of abundance of other groups will be less precise. Biomass (mg wet weight/m^3^) was calculated using individual species‐ and stage‐specific weights according to Hernroth (1985). For conversion to dry weight (mg dry weight/mg wet weight), a factor of 0.20 was used according to the ICES Zooplankton Methodology Manual (see table 4.7 in Page 141; Postel, Fock, & Hagen, 2000).

For further analyses of total lipids and its wax ester class, total proteins, individual FA and AA composition, bulk and compound‐specific δ^13^C isotope values in FAs and AAs, and additional daytime and nighttime samples of zooplankton from 3 to 4 casts from all depths intervals (e.g., SW, WW, and BW) were collected and separated into two size classes of 100–300 µm and ≥300 µm using a sieve with a mesh size of 300 µm. Before size fractionation, mesozooplankton from the surface mixed water layer was concentrated in a light trap for 3 hr to avoid sample contamination with phytoplankton cells. On board, the size fractions were subsequently shock‐frozen in liquid N_2_ and stored at −80°C. In the laboratory, all deep‐frozen samples were lyophilized for 48 hr under vacuum (Fudge, 1968). Freeze‐dried mesozooplankton was ground to a fine powder. Homogenized mesozooplankton were stored at −80°C for later concentration and stable isotope analyses.

### 2.2 Fatty acid and amino acid composition analysis

Analysis of FAs trophic markers is a quantitative approach that can provide information on the integrated dietary sources in consumers on time scales of several weeks (Auel, Harjes, Rocha, Stübing, & Hagen, 2002; Boissonnot, Niehoff, Hagen, Søreide, & Graeve, 2016). For example, high content of essential EPA in zooplankton usually is associated with a diatom‐based diet (Graeve, Hagen, & Kattner, 1994; Volkman, Jeffrey, Nichols, Rogers, & Garland, 1989), while a high content of DHA is used predominantly as a marker for (dino‐) flagellates (Dalsgaard et al., 2003; Graeve et al., 1994). In the Baltic Sea 18:2(n‐6) and 18:3(n‐3), FAs are usually considered as indicative biomarkers for cyanobacteria (Gugger et al., 2002; Peters et al., 2013, 2006; Vargas et al., 1998). It must be pointed out that trace levels of specific FA trophic markers of a particular algae class still can be found in other algae classes as well (Dalsgaard et al., 2003).

The extraction of lipids and separation of individual FAs (trophic markers) and FAlc of mesozooplankton were conducted at the Alfred Wegener Institute, Germany. Lipids from freeze‐dried samples were extracted using a modified procedure from Folch, Lees, and Stanley (1957) with dichloromethane/methanol (2:1, v/v) and a washing procedure with an aqueous KCl solution (0.88%). For quantification of FAs, tricosanoic acid (23:0) was added as an internal standard prior to extraction. The lipid extract was redissolved in dichloromethane and taken for analysis or kept at −20°C for further analyses. For the gas–liquid chromatography analyses of FAs and FAlc, the aliquots of the total lipid extract were taken. The total lipid extracts were hydrolyzed under nitrogen atmosphere in methanol containing 3% concentrated sulfuric acid and were transesterificated for 4 hr at 80°C. After a subsequent cyclohexane extraction, the resulting fatty acid methyl esters (FAMEs) and alcohols were separated with an Agilent 6890N Network gas chromatograph (GC, Agilent Technologies) on a 30‐m wall coated open tubular DB‐FFAP column (0.25 mm I.D., film thickness: 0.25 μm) equipped with a split injection and a flame ionization detector using a temperature program according to Kattner and Fricke (1986). Helium was used as a carrier gas. FAMEs and alcohols were identified by comparing the retention time with commercially available standard mixtures and quantified with an internal standard.

Additionally, the wax ester lipid class in mesozooplankton from the EGB was analyzed. Wax esters were not separated chromatographically, but calculated as follows: Total lipids were calculated using the sum of the measured 26 FAs and 2 FAlc and were expressed as a percentage of dry weight (% DW). The percentage of wax esters in total lipid was calculated from the proportion of alcohols on a mole basis, assuming that copepods contain no free fatty alcohol (Kattner & Fricke, 1986; Kattner & Krause, 1989). The wax esters were expressed as % of total lipids (% TL). The proportions of individual FAs and FAlc were expressed as % of total FA and FAlc, respectively. The individual FAs of mesozooplankton were grouped into saturated fatty acids (SFAs), monounsaturated fatty acids (MUFAs), and polyunsaturated fatty acids (PUFAs).

Derivatization of individual AAs in POM and mesozooplankton samples was performed at the Leibniz Institute for Baltic Sea Research Warnemuende (IOW), Germany. Freeze‐dried POM filters and mesozooplankton subsamples were prepared for GC analysis by acid hydrolysis followed by derivatization to trifluoro‐acetylated isopropyl amino acid esters (AA‐TFA/IP) following the standard protocols as in Eglite et al. (2018) and Hofmann, Gehre, and Jung (2003) including a clean‐up step with chloroform:phosphorus buffer (KH_2_PO4 + Na_2_HPO_4_ in Milli‐Q water, pH 7) in ratio 1:2 as described by Veuger, Middelburg, Boschker, and Houtekamer (2007). To determine the recovery of AAs, the tranexamic acid was added as an internal standard to each sample prior to the acid hydrolysis. Each time an aliquot and external standard with 16 commercially available AAs (containing 0.1 µg per µl of individual AA) were derivatized together with the new sample set. The standard mixture included alanine (Ala), arginine (Arg), aspartic acid (Asp), cysteine (Cys), glutamic acid (Glu), glycine (Gly), isoleucine (Ile), leucine (Leu), lysine (Lys), methionine (Met), phenylalanine (Phe), proline (Pro), serine (Ser), threonine (Thr), tyrosine (Tyr), and valine (Val). During AA hydrolysis, the amide bond in glutamine (Gln) and asparagine (Asn) is cleaved and converted to Glu and Asp, respectively; therefore, these AAs are determined as the combined peak of Glu and Asp in the chromatogram (Davidson, 1997). The prepared AA‐TFA derivatives were measured using a DANI gas chromatograph (DANI, Master GC, Italy) equipped with a RXI‐5Sil MS column (40 m, 0.18 mm I.D., film thickness: 0.18 µm, Restek GmbH) and a DANI mass selective time of flight detector (DANI TOF MS Plus). Injections were done via a programmed temperature vaporizing (PTV) injector. Helium was used as a carrier gas. An external standard was processed in parallel with the samples for quantitative comparison to each serial of analyses. The individual AAs in samples were identified by comparison of the retention times and mass spectra with those of the standard mixture. In our samples, Arg, Cys, and His sometimes were below the limit of detection; thus, these AAs were excluded. Due to the low yield of Met during the derivatization, the TFA‐Met product was also excluded from the further interpretations (Hofmann et al., 2003). For more details on the derivatization method, the sample preparation, and the temperature program, see Eglite et al. (2018).

The total protein content was acquired as an amount of total hydrolysable amino acids (THAAs) after summing up 15 individual AAs (except His) and expressed as % of DW. Individual AAs were expressed as % of THAAs in comparison with the FA and FAlc pools. Additionally, individual AAs were grouped into nonessential and essential ones, according to a classification for crustaceans (Claybrook, 1983).

### 2.3 Bulk and compound‐specific stable isotope analyses

The quantitative investigations of diet‐consumer relationships can be complemented by tools for elucidating dietary carbon sources via bulk and compound‐specific δ^13^C analyses of essential FAs and AAs. In addition, the δ^13^C patterns of nonessential compounds that can be synthesized de novo by animals can be used to construe detailed information about internal biosynthesis processes but also reflect the isotopic signatures from dietary sources (McMahon et al., 2010).

Bulk δ^13^C values in POM and mesozooplankton samples were determined by an Elemental Analyzer (EA, Thermo Flash EA 1112) coupled to a continuous‐flow isotope ratio mass spectrometer (IRMS, Thermo Finnigan Delta^Plus^) via an open split interface (Thermo Finnigan Conflo III) at IOW. The internal laboratory reference gas was ultrapure carbon dioxide, which was calibrated against the materials from the International Atomic Energy Agency (IAEA): NBS 22 (mineral oil δ^13^C = −29.74‰) and USGS 24 (graphite δ^13^C = −15.99‰). The relative differences from the corresponding standard reference material (*R*_reference_), which is certified Vienna Pee Dee Belemnite (PDB) carbon, are reported as delta (δ) notation in units of parts per thousand (‰) as follows: δ*X* (‰) = [(*R*_sample_/*R*_reference_) − 1)] × 10^3^, where *X* is the δ^13^C, and *R*_sample_ is the ^13^C:^12^C ratio in a sample. In addition, peptones (Merck) were analyzed as in‐house standards after every sixth mesozooplankton sample run. The analytical error for stable isotope ratios indicated by the peptone standards was less than ±0.2‰ for carbon isotopes. All samples were analyzed in duplicates.

The δ^13^C composition in FAMEs was measured for the most abundant FAs in mesozooplankton samples using a Thermo GC‐C‐IRMS system, equipped with a Trace GC Ultra gas chromatograph, a GC IsoLink, and a Delta V Plus isotope ratio mass spectrometer, connected via a Conflo IV interface (Thermo Scientific Corporation). The FAMEs, dissolved in hexane, were injected in splitless mode and separated on a 60 m long DB‐FFAP column (0.25 mm I.D., film thickness: 0.25 μm; liquid phase). The performed temperature program is described by Kohlbach et al. (2016). The δ^13^C values of individual FAMEs were calibrated by analyzing the certified standard FAMEs 14:0 and 18:0 (Indiana University) after every fifth sample. The analytical error was ±0.3‰ for both standards. Furthermore, for reproducibility and analytical precision of the isotope measurements, the laboratory standard 23:0 was measured during the sample runs with an analytical error of ±0.4‰. The samples were analyzed in duplicates.

During the transesterification, the added methyl group can slightly change the δ^13^C values of FAMEs compared to free FAs (Wang, Budge, Gradinger, Iken, & Wooller, 2014). The latest study by Kohlbach et al. (2016) reported insignificant δ^13^C differences. Thus, for our samples the corrections for derivatization were not applied.

The δ^13^C composition in AA‐TFA derivatives was determined by a Thermo MAT 253 isotope ratio mass spectrometer (IRMS) coupled to a Thermo Trace GC 1310 gas chromatograph (GC) fitted with a BPX‐5 (60 m, 0.32 μm I.D., film thickness: 1.0 μm, SEG Analytical Science) via a ConFlo IV interface (all parts from the Thermo Fisher Scientific GmbH). The combustion unit was a Thermo IsoLink fixed at the GC oven and connected to the IRMS via a ConFlo IV interface. The injection of the AA‐TFAs, which were dissolved in dichloromethane, was done via a PTV injector in splitless mode and helium was used as carrier gas. The temperature program was used as described in Eglite et al. (2018). The external standard with 16 AAs was treated in parallel with each sample run to confirm the reproducibility of isotope measurements. The precision of our isotopic measurements varied among size fractions, but the standard deviation of triplicate measurements typically was on the order of <1‰. In summary, we determined δ^13^C values of 10 AAs, except for Arg, Cys, Met, Ser, Val, and Tyr, due to their small amounts or poor chromatographic separation of individual peaks in half of the mesozooplankton samples.

During the derivatization, the added carbon by the isopropyl and N‐trifluoroacetyl groups might have an effect on the δ^13^C values of the AAs carbon skeleton (Docherty, Jones, & Evershed, 2001; Silfer, Engel, Macko, & Jumeau, 1991). Here, the reported δ^13^C values of TFA‐AA were compared to the external AA standard mixture with known stable carbon isotopic composition and were corrected after Silfer et al. (1991). The analytical error of internal standard tranexamic acid was ±0.4‰. The daily external standard typically was better than 0.7‰ for AAs presented in this study.

### 2.4 Calculations and statistical analyses

All data in tables and figures are given as mean ± standard deviation from at least two samples, which were sampled during daytime and nighttime hours. Differences between mesozooplankton communities, size fractions, two summers, and difference between compound‐specific δ^13^C values and bulk δ^13^C values in POM were tested by one‐way and two‐way analyses of variance (ANOVA, *α* = 0.05) using Excel. Additionally, we conducted two principal component analyses (PCAs) including δ^13^C data from essential AA to identify (a) trophic connections between POM and mesozooplankton at the EGB and (b) spatial differences in the δ^13^C signatures of mesozooplankton across the five different sub‐basins (WB, AB, BB, SGB, and EGB; Figure 1) of Baltic Sea. The PCAs were done using PRIMER‐6 Software (Primer‐E Ltd.). Then, we applied a simple plot of the three most informative AAs according to Larsen et al. (2009). Differences in the δ^13^C values between the essential AAs Ile and Leu (Δδ^13^C_Ile‐Leu_) and Ile and Lys (Δδ^13^C_Ile‐Lys_), respectively, are considered to be most informative for dietary end‐member identification. Therefore, we compared the Δδ^13^C_Ile‐Leu_ and Δδ^13^C_Ile‐Lys_ of our POM samples from the EGB with Δδ^13^C values of bacteria and microalgae from Larsen et al. (2009), Larsen et al. (2013). Further, to better explain the origin of FA 18:1(n‐9) in our mesozooplankton, we applied a linear regression analysis with an independent proxy for the incorporation of detrital material by mesozooplankton, the δ^15^N‐AA based heterotrophic resynthesis index ΣV (McCarthy, Benner, Lee, & Fogel, 2007). The ΣV data were acquired for the same mesozooplankton samples as used in this study from Eglite et al. (2018). Additionally, we tested whether the enrichment of δ^13^C in 18:1(n‐9) was linked to an increase in wax ester content using linear regression (Falk‐Petersen, Hagen, Kattner, Clarke, & Sargent, 2000).

### 2.5 Mesozooplankton feeding mode in the field

In order to explain the flow of dietary C in the upper and deeper water column communities linked to their respective feeding mode, we acquired TP estimates of the different mesozooplankton communities from three water bodies (SW, WW, and BW) at the EGB from Eglite et al. (2018) and included the data in Table 2. TP estimates were based on the difference in δ^15^N values of Glu and Phe with a given uncertainty of ±0.3 (Chikaraishi et al., 2014). The TP estimates revealed mainly omnivorous and carnivorous feeding modes (TP > 2.5) in the SW and the WW communities dominated by cladocerans and *T. longicornis* in 2014 and 2015, respectively (Eglite et al., 2018). In the BW, the mesozooplankton dominated by *Pseudo‐/Paracalanus* spp. appeared to be solely carnivore (TP of 3.0 ± 0.1). Results by Eglite et al. (2018) indicated an absence of a clear herbivore signal (TP of 2.0) in both summers.

## 3 RESULTS

### 3.1 Environmental and biological data

During the two summers, the water column was stratified and separated by the thermocline and halocline, thus, creating three distinct water bodies that mainly differed in hydrographic conditions (Table 1). A major difference between both summers was in sea surface temperature (SST) in the surface mixed water layer. In 2014, SST of 19.8°C was considerably higher compared to SST of 16.2°C in 2015. Below the thermocline, the temperature ranged between 3.5°C and 5.5°C in the WW during both summers, which reflects the conditions of the water column from the preceding winter (Leppäranta & Myrberg, 2009). In the BW below the halocline, the temperature of around 6°C was slightly higher than in the WW during both years. Salinity ranged between 6.7 and 7.6 throughout the UWC (incl. SW and WW: 0–max. 65 m) but increased up to 11.7 below the halocline in the BW.

**Table 1:** Hydrographic parameters, mesozooplankton biomass in dry weight (mg DW/mg WW), and percentage (%, in brackets) including all development stages of main taxonomic groups and dominant species in total biomass from three water bodies at the Eastern Gotland Basin in July 2014 and 2015

	Surface water		Winter water		Bottom water	
	2014	2015	2014	2015	2014	2015
Depth	0−10 m	0−25 m	10−60 m	25−65 m	60−125 m	65−110 m
Temperature (°C)	17.9–19.8	8.0–16.2	3.5–17.9	4.4–8.0	3.5–5.6	7.4–11.4
Salinity	6.6–6.8	6.8–7.2	6.6–7.5	7.5–8.4	4.5–6.3	8.4–11.7
Copepods	26,598 (34)	**62,119 (86)**	**83,558 (94)**	**91,989 (99)**	**12,147 (97)**	**1,154 (99)**
*Temora longicornis*	2,202 (3)	**42,864 (59)**	**62,110 (70)**	**68,875 (74)**	206 (2)	1,038 (9)
*Acartia* spp.	20,861 (26)	4,596 (6)	4,541 (5)	3,078 (3)	56 (<1)	62 (1)
*Centropages* spp.	372 (<1)	12,753 (18)	3,711(4)	13,488 (14)	30 (<1)	236 (2)
*Pseudo/Paracalanus* spp.^a^	761 (<1)	280 (0)	5,964(7)	6,458 (7)	**11,744 (94)**	**10,187 (88)**
Cladocera	**43,743 (56)**	4,636 (6)	3,782 (4)	715 (1)	62 (<1)	
*Bosmina* spp.	**30,440 (39)**	116 (<1)	219 ( <1)	–	26 (<1)	
*Evadne nordmani*	251 (<1)	1792 (2)	3,249 (4)	560 (<1)	17(<1)	
*Pleopsis polyphemoides*	9,165 (12)	1984 (3)	313 (0)	155 (<1)	19 (<1)	
*Podon intermedius*	3,610 (5)	744 (1)	–	–	–	–
Rotifera	8,380 (11)	5,407 (7)	1,324 (1)	379 (<1)	45 (<1)	6 (<1)
*Synchaeta*	8,026 (10)	5,379 (7)	1,321 (1)	379 (<1)	45 (<1)	6 (<1)
*Keratella*	355 (<1)	28 (<1)	2 (<1)	–	–	–

Notes

Bold values indicate most abundant taxa and species during the sampling period.

Total biomass includes the taxonomic groups of balanus, polychaeta, bivalvia, gastropoda, copelata.

a can include individuals of *Paracalanus* spp. due to uncertainties in the identification of younger stages of this genus.

The difference in SST changed the mesozooplankton composition (Eglite et al., 2018) and biomass between both summers. In the warmer summer of 2014, the biomass of mesozooplankton comprised mainly of cladocerans of the genus *Bosmina* spp., which contributed 39% of the total biomass (30,440 mg DW/mg WW), followed by copepods of *Acartia* spp. (26% or 20,861 mg DW/mg WW) in the SW community (Table 1). Nevertheless, *T. longicornis* was a dominant copepod species throughout the UWC, but was submerged in the colder intermediate WW during 2014, where it formed 70% (62,110 mg DW/mg WW) of the total mesozooplankton biomass in. In contrast, in the colder summer of 2015, *T. longicornis* biomass considerably increased and dominated also the SW (59% or 42,854 mg DW/mg WW), while its relative biomass in the WW (74% or 68,875 mg DW/mg WW) was similar to 2014. With depth, we observed a shift in the dominant copepod species from temperate *T. longicornis* in the UWC to boreal *Pseudo‐/Paracalanus* spp. in the BW. The total biomass of *Pseudo‐/Paracalanus* spp. was 11,744 mg DW/mg WW and 10,187 mg DW/mg WW in 2014 and 2015, respectively, which is equivalent to 88%–94% of the total biomass of mesozooplankton in the BW during both years. However, compared to *T. longicornis* in the UWC, the absolute biomass of *Pseudo‐/Paracalanus* spp. was still approximately 5–6 times lower.

### 3.2 Lipid and protein content

In two summers, the total lipid content in mesozooplankton size fractions ranged from minimum ~ 2% to maximum ~ 38% of DW and mainly varied between communities dominated by either *T. longicornis* or *Pseudo‐/Paracalanus* spp. (Tables 1 and 2). The total lipid content of the mesozooplankton size fractions from the SW was always about a twofold lower compared to the community from the WW (one‐way ANOVA *F*_7.5_ = 4.4, *p* < .01) in both summers. Interestingly, the total lipid content in the SW remained similar during both years independent of whether the mesozooplankton population was dominated by the cladocerans *Bosmina* spp. or by the copepod *T. longicornis* (Tables 1 and 2). In contrast, the *Pseudo‐/Paracalanus* spp.‐dominated community from the BW was significantly enriched in lipids compared to the communities from the UWC (in 2014, one‐way ANOVA *F*_48.3_ = 5.1, *p* < .001). Specifically, during both years the average total lipid content in mesozooplankton size fractions remained in the relatively narrow range of 2.8%–4.7% of DW in the SW, then slightly increased to a range of 6.9%–10.4% of DW in the WW, while the mesozooplankton from the available data set from the BW in 2014 reached the highest total lipid values of 25.0%–28.2% of DW (Table 2).

**Table 2:** Total lipid and total protein content (% of dry weight), wax esters (% of total lipids), composition of fatty alcohols (% of total fatty alcohols), and trophic positions according to Eglite et al. (2018) in two mesozooplankton size fractions from isolated water bodies (BW, bottom water; SW, surface water; WW, winter water) at the Eastern Gotland Basin in July 2014 and 2015. Note that mesozooplankton from the BW was sampled only in 2014

	2014						2015			
	SW	SW	WW	WW	BW	BW	SW	SW	WW	WW
	0−10 m	0−10 m	10−60 m	10−60 m	65−125 m	65−125 m	0−25 m	0–25 m	25−65 m	25−65 m
Size fraction (μm)	100–300	≥300	100–300	≥300	100–300	≥300	100–300	≥300	100–300	≥300
	*(n* = 2)	(*n* = 1)	(*n* = 2)	(*n* = 2)	(*n* = 2)	(*n* = 2)	(*n* = 4)	(*n* = 3)	(*n* = 4)	(*n* = 3)
Total lipids	3.8 ± 0.6	3.9	6.9 ± 3.7	9.7 ± 6.0	25.0 ± 0.8	28.2 ± 9.5	4.7 ± 2.0	2.8 ± 0.8	9.2 ± 2.8	10.4 ± 10.3
Total proteins	43.4 ± 1.5	47.4 ± 0.8	42.5 ± 3.9	47.6 ± 8.4	29.6 ± 0.5	25.1 ± 1.6	38.4 ± 8.5	49.6 ± 9.3	30.6 ± 4.7	41.4 ± 1.6
Wax esters	–	–	35.1 ± 28.5	34.3 ± 28.1	72.8 ± 4.3	66.3 ± 0.7	12.7 ± 20.3	1.0 ± 1.8	43.2 ± 12.2	25.3 ± 26.2
Fatty alcohol										
14:0	–	–	30.2 ± 1.2	30.4 ± 0.7	31.1 ± 0.1	30.4 ± 0.1	57.0 ± 30.0	43.8	35.2 ± 1.4	37.3 ± 0.2
16:0	–	–	69.8 ± 1.2	69.6 ± 0.7	68.9 ± 0.1	69.6 ± 0.1	57.3 ± 10.8	56.2	64.8 ± 1.4	62.7 ± 0.2
TP_Glu/Phe_^*^	2.5 ± 0.1	25	2.5 ± 0.3	2.6 ± 0.1	2.9 ± 0.0	3.0 ± 0.1	2.5 ± 0.3	2.9 ± 0.0	2.9 ± 0.0	2.8 ± 0.1

In two summers, the total protein content in mesozooplankton size fractions ranged from minimum ~ 18% to maximum ~ 59% of DW. Similar to lipids, the total protein content differed between the UWC and the BW communities dominated by either *T. longicornis* or *Pseudo‐/Paracalanus* spp. (Tables 1 and 2). Protein content was significantly higher in mesozooplankton size fractions from the UWC compared to the BW community (in 2014, one‐way ANOVA *F*_8.6_ = 5.3, *p* < .01). No significant difference in the total protein content was found for mesozooplankton size fractions from the SWs and the WWs between both summers (one‐way ANOVA *F*_4.30_ = 1.40, *p* = .25) or among water layers (one‐way ANOVA *F*_4.30_ = 0.12, *p* = .79). Specifically, the averaged protein content in mesozooplankton size fractions ranged from 30.6% to 49.6% of DW throughout the UWC during both years, while in the BW community, the total protein content was 25.1%–29.6% of DW in 2014 (Table 2).

### 3.3 Wax esters

Wax esters were the dominant lipid class in the mesozooplankton size fractions from the BW, where they accounted for 66.3%–72.8% of total lipids (Table 2). Relatively high levels of wax esters accounting for 25.3%–43.2% of total lipids were also determined in the mesozooplankton size fractions from the WW in both years. In contrast, wax esters were either absent or very low with max. 12.7% of total lipids (Table 2) in mesozooplankton size fractions from the SW in 2014 and 2015. Notably, during both years we observed high standard deviations (*SD*s) of wax ester content in both mesozooplankton size fractions, namely from the WWs (±28.1%–28.1% of total lipids and ±12.2%–26.2% of total lipids in 2014 and 2015, respectively, in Table 2). During both years, we observed a substantial increase in wax ester content during nighttime hours (max 55.3% and 61.3% of total lipids in 2014 and 2015, respectively; Appendix S1: Table S1) compared to daytime hours (max 14.9% and 38.2% of total lipids in 2014 and 2015, respectively; Appendix S1: Table S1), namely in animals from the WW.

### 3.4 Composition of individual compounds: fatty acids, fatty alcohols, amino acids

**Figure 2:**
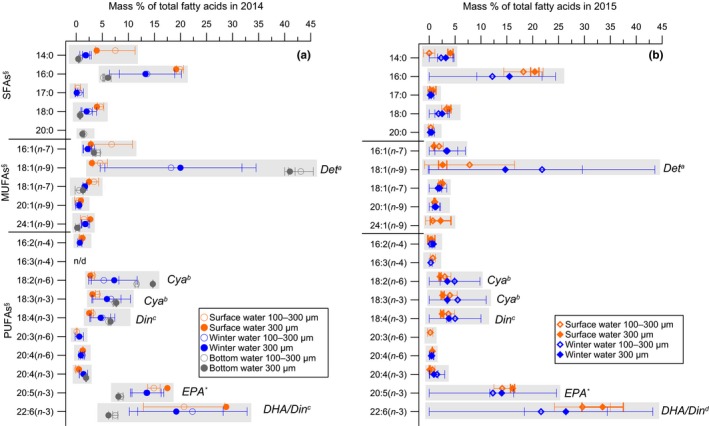
Composition of fatty acids (% of total fatty acids) in two mesozooplankton size fractions (100–300 µm and 300 µm) from three water bodies separated by the vertical water column stratification at the Eastern Gotland Basin in July 2014 (a) and 2015 (b). *Notes*: The bottom water was collected only in 2014. Abbreviations: DHA, docosahexaenoic acid; EPA, eicosapentaenoic acid; MUFAs, monounsaturated fatty acids; PUFAs, polyunsaturated fatty acids; SFAs, saturated fatty acids. Dietary trophic markers: *Det^a^*—detritus (Kattner & Krause, 1989; Peters et al., 2006); *Cya^b^*—cyanobacteria (Peters et al., 2013; Vargas et al., 1998); DHA/Din^c^—dinoflagellates (Dalsgaard et al., 2003; Graeve et al., 1994), EPA^*^—typical for diatoms (Graeve et al., 1994; Volkman et al., 1989), but together in high quantities with DHA signals the presence of dinoflagellates (Ahlgren et al., 1992; Mansour et al., 1999)

In all samples, the FA composition was dominated by (a) shorter‐chain SFAs (namely 14:0, 16:0, 18:0); (b) MUFAs of varying length (namely 16:1(n‐7), 18:1(n‐7), 18:1(n‐9), and 24:0(n‐9); and (c) of six long‐chain PUFAs (namely 18:2(n‐6), 18:3(n‐3), 18:4(n‐3), 20:4(n‐3), 20:5(n‐3), or EPA, 22:6(n‐3) or DHA; Figure 2a,b). Among all FA classes, PUFAs had the highest content (mass % of total FAs) in all communities (Figure 2a,b, Appendix S1: Table S2). EPA and DHA represented two major PUFAs in our mesozooplankton samples (Figure 2a,b). Significantly higher EPA and DHA contents were measured in the UWC communities compared to mesozooplankton from the BW in 2014 (one‐way ANOVA for EPA *F*_5.11_ = 25.5, *p* < .001; for DHA *F*_5.11_ = 16.1, *p* < .01, Figure 2a,b, Appendix S1: Table S2). We also found no significant difference in EPA and DHA content in mesozooplankton size fractions from the SWs and the WWs between both summers (one‐way ANOVA for EPA *F*_4.35_ = 0.25, *p* = .62; for DHA *F*_4.35_ = 3.09, *p* = .09). Additionally, the three PUFAs 18:2(n‐6), 18:3(n‐3), and 18:4(n‐3) were also found in high quantities throughout all communities (Figure 2a,b). These FAs had similar content ranges in mesozooplankton size fractions from the UWC (Figure 2a,b; Appendix S1: Table S2) and showed a significant increase (except for 18:3(n‐3)) in the BW community in 2014 (UWC vs. BW one‐way ANOVA for 18:2 (n‐6) *F*_5.11_ = 26.5, *p* < .001; for 18:4 (n‐3) *F*_5.11_ = 7.84, *p* < .05; for 18:3(n‐3) *F*_5.11_ = 3.73, *p* = .09). In the BW community that was dominated by *Pseudo‐/Paracalanus* spp., the content of FA 18:1(n‐9) was significantly higher than in the UWC community that was dominated by *T. longicornis* (one‐way ANOVA *F*_5.11_ = 24.8, *p* < .001; Figure 2a, Appendix S1: Table S2). Notably, the 18:1(n‐9) content had relatively high variations in both mesozooplankton size fractions from the WW compared to the animals from the other water bodies (based on SDs of mean value ±13.6%–14.5% and ±7.9%–14.9% of total FAs in 2014 and 2015, respectively; Appendix S1: Table S2). The FAlc composition of the Baltic Sea mesozooplankton communities consisted only of short‐chain saturated moieties such as 14:0 and 16:0. Interestingly, FAlcs were below the detection limit in the SW at times of cladocerans dominance in 2014 (Table 2).

The AA composition and content (mass % of THAA) of nonessential and essential compounds in *T. longicornis* and *Pseudo‐/Paracalanus* spp.‐dominated communities were similar throughout both size fractions and the two summers (Appendix S1: Table S3 and Figure S1). We compared the mass % of THAA content of individual AAs with the mean AA values of *T. longicornis* from Guisande, Maneiro, Riveiro, Barreiro, and Pazos (2002). The average AA values of both, the UWC and the BW, communities in our study at times of intense cyanobacterial blooms resembled the AA results of *T. longicornis* that had been feeding on dinoflagellates and diatoms in the absence of cyanobacteria (Appendix S1: Figure S1).

### 3.5 Bulk and compound‐specific carbon stable isotope patterns

**Figure 3:**
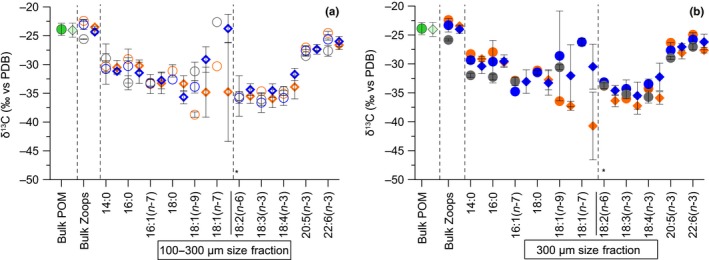
δ^13^C values (mean ± *SD*) of bulk in particulate organic matter (POM) and two mesozooplankton size fractions ((a) 100–300 µm; (b) ≥300 µm), and in individual fatty acids (in ‰ vs. PDB) only in mesozooplankton from the surface water (orange symbols), the winter water (blue symbols), and the bottom water (gray symbols) at the Eastern Gotland Basin in July 2014 (circles) and 2015 (diamonds). *Notes*: Error bars are standard deviations over a 24‐hr sampling period of mesozooplankton (*n* = 2–4 from the particular depth of each size fraction). POM was pooled from different depths (from 10 m to max. 100 m, bright green, *n* = 6) in 2014, and from the SW and the WW (from 1 m to max. 30 m, pale green, pooled sample, *n* = 8) in 2015. (*)—Separation between nonessential (left) and essential (right) component

**Figure 4:**
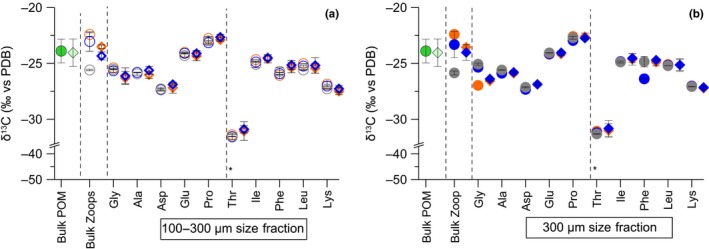
δ^13^C values (mean ± *SD*) of bulk in particulate organic matter (POM) and two mesozooplankton size fractions ((a) 100–300 µm; (b) ≥300 µm), and in individual amino acids only in mesozooplankton from the surface water (orange symbols), the winter water (blue symbols), and the bottom water (gray symbols) at the Eastern Gotland Basin in July 2014 (circles) and 2015 (diamonds). See notes in Figure 3

We compared bulk δ^13^C values of POM with essential FAs and AAs δ^13^C values of mesozooplankton samples to evaluate which FAs and AAs of consumers reflected δ^13^C values of POM. In mesozooplankton, the δ^13^C values of the two essential FAs EPA (20:5(n‐3)) and DHA (22:6(n‐3)) were similar by a difference of 2–4‰ to the bulk δ^13^C values of POM, while essential FAs with 18C were consistently depleted by 10‰ (Figure 3a,b). In contrast, almost all essential AAs in mesozooplankton had δ^13^C values that were close to the bulk δ^13^C values in POM in both years, except for Thr, which was depleted by around 6‰. More specifically, the δ^13^C values of Ile, Phe, and Leu in mesozooplankton were depleted by around 1–2‰, while Lys was depleted by around 3‰ compared to the bulk δ^13^C values in POM (Figure 4a,b). Although the differences in δ^13^C values between POM and two FAs and three AAs in mesozooplankton were small, they were still significant (two‐way ANOVA for POM vs. 20:5(n‐3) and 22:6(n‐3) from both years *F*_4.24_ = 52.5, *p* < .001; two‐way ANOVA for POM vs. Ile, Phe, Leu from both years *F*_3.17_ = 38.8, *p* < .001).

We compared δ^13^C patterns of both essential and nonessential FA and AA pools between mesozooplankton size fractions and between the two summers dominated by *T. longicornis* and *Pseudo‐/Paracalanus* spp. (Table 1). Overall, the compound‐specific δ^13^C values of FAs covered a broad range of values from −22‰ to as low as −44‰ (Figure 3a,b). The δ^13^C values in essential FAs were conservative according to minor SDs changes in both mesozooplankton size fractions, while considerable variations in the δ^13^C values were found in some nonessential FAs according to large *SD* ranges (Figure 3a,b). The largest *SD*s of δ^13^C mean values were found for the nonessential FAs 18:1(n‐9), for example, ±7.7‰ (*SD*) and 18:1(n‐7), for example, ±8.6‰ (*SD*) from both mesozooplankton size fractions (Figure 3a,b). In contrast to other nonessential FAs, the averaged mean values of 18:1(n‐9) and 18:1(n‐7) scattered the most between mesozooplankton communities (Figure 3a,b), whereas the exclusively dominated BW community by *Pseudo‐/Paracalanus* spp. (Table 1) had much more enriched δ^13^C values of 18:1(n‐9) of around −30‰ (Appendix S1: Table S1, mesozooplankton from 65 to 125 m). The δ^13^C values in the AAs were in a comparatively smaller range than the FAs (−22‰ to −27‰; Figure 4a,b). In contrast, the SDs variations of mean δ^13^C‐AA values were considerably smaller among both essential and nonessential AAs, that is, max. ±0.7‰ (*SD*) in each pool. Further, we did not observe any distinctive pattern between mesozooplankton communities from the different depth layers or size fractions in either year (Figure 4a,b).

### 3.6 Content and δ^13^C changes in the FA 18:1 (n‐9) in mesozooplankton

**Figure 5:**
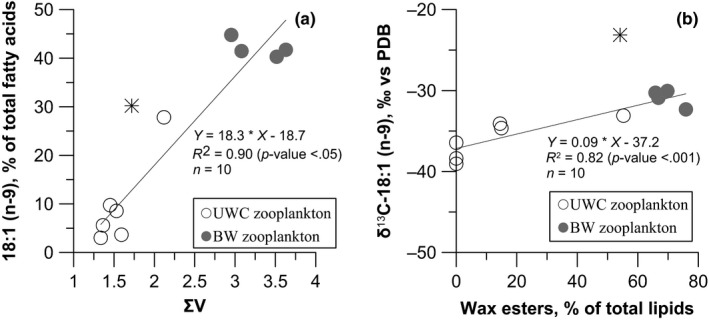
(a) The fatty acid 18:1 (n‐9) content (% of total fatty acids) of mesozooplankton size fractions relative to microbial degradation index (Σ) results from Eglite et al. (2018). (b) The δ^13^C values of fatty acid 18:1 (n‐9) relative to the wax ester content (% of total lipids). The statistics for the respective regression are included in the figures.* Notes*: The linear regression analysis was used to quantify the relationship between the upper water column (UWC, open circles) and the bottom water (BW, gray circles) mesozooplankton communities. One data point (from the UWC in 2014 at 23:15 hr, a mixing point with *Pseudo‐/Paracalanus* spp. community, Appendix S1: Table S1) was excluded from both regression analyses

To better understand the variations of 18:1(n‐9) content (mass % of total FAs) in mesozooplankton samples, we applied a linear regression analysis with results of microbial degradation index ΣV (Eglite et al., 2018). After excluding a single outlier from the data set from 2014, a significant positive correlation between the ΣV and the 18:1(n‐9) content from both mesozooplankton size fractions was found, mainly due to the differences between the UWC and the BW communities (*R* ^2^ = .90, *p* < .05; Figure 5a). Further, the enrichment of δ^13^C values in 18:1(n‐9) was positively correlated with an increase in wax ester content (% of total lipids) in the *Pseudo‐/Paracalanus* spp.‐dominated mesozooplankton community from the BW in 2014 (*R* ^2^ = .82, *p* < .001; Figure 5b).

### 3.7 Analysis of essential amino acids δ^13^C patterns

**Figure 6:**
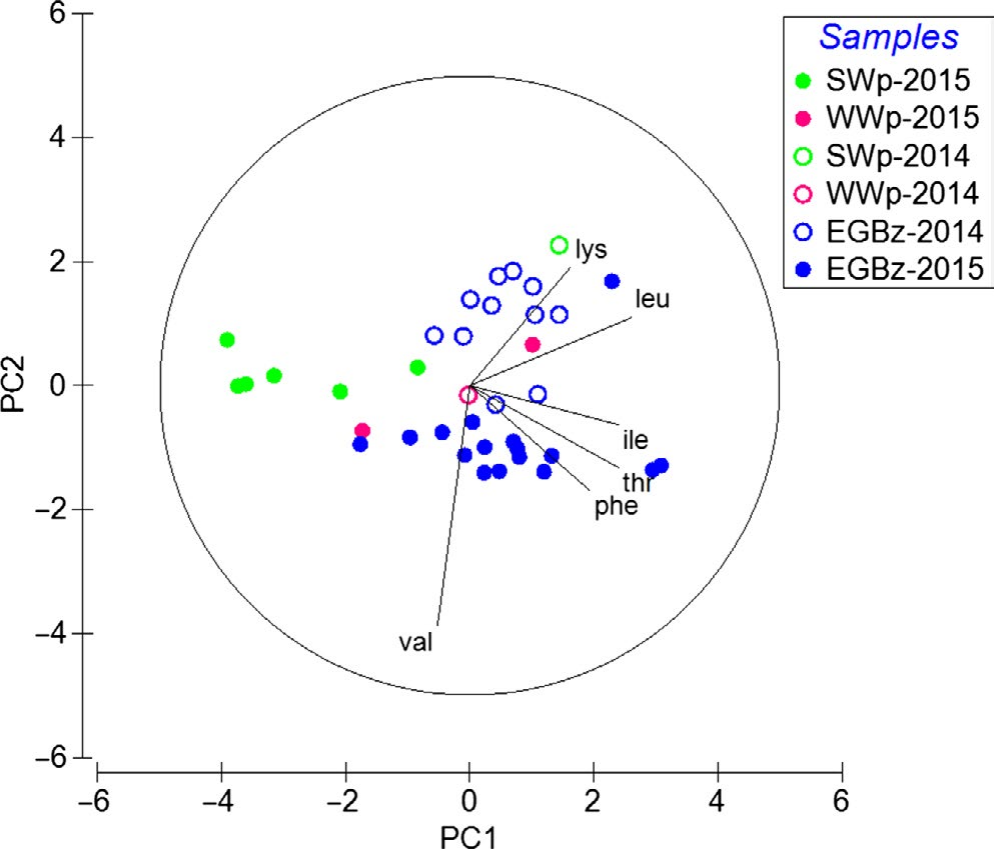
The principal component analysis of δ^13^C values in essential amino acids of particulate organic matter from the surface water (SWp, green circles) and the winter water (WWp, pink circles) together with mesozooplankton of the overall water column (0–max. 125 m) at the Eastern Gotland Basin (EGBz, blue circles) from two summers in July 2014 (open symbols) and 2015 (filled symbols). *Notes*: Together, the two leading principal components (PCs) explained 67.8% of the variability, with PC1 and PC2 accounting for 47.7% and 20.7% of the variability by each axis, respectively. The amino acids are indicated by their three‐letter amino acid code as explained in the text

**Figure 7:**
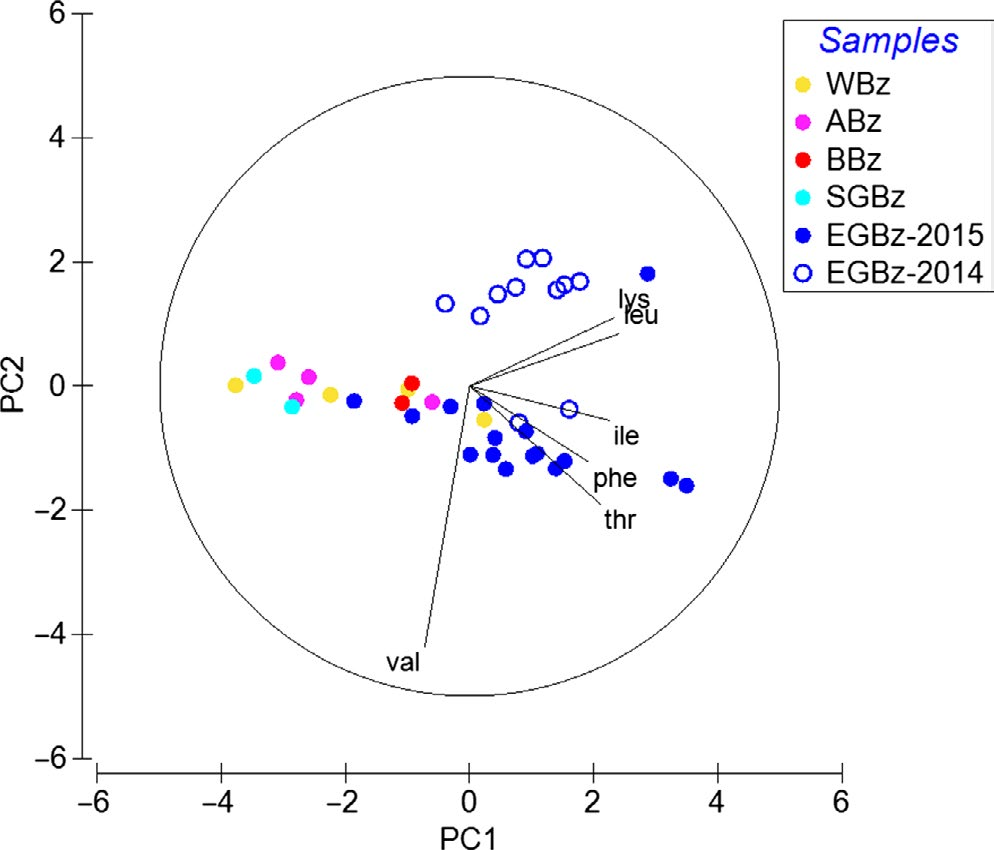
The principal component analysis of δ^13^C values in essential amino acids of mesozooplankton samples from the five sub‐basins of the Baltic Sea in 2015, including those from the Eastern Gotland Basin. *Notes*: Together, the two leading PCs explained 74.2% of the variability, with PC1 and PC2 accounting for 54.4% and 19.8% of the variability, respectively. Abbreviations of sub‐basins: AB, Arkona Basin (pink circles); BB, Bornholm Basin (red circles); EGB, Eastern Gotland Basin (blue circles); SGB, Southern Gotland Basin (cyan circles); WB, Western Baltic (yellow circles). The amino acids are indicated by their three‐letter amino acid code and explained in the text

Two PCAs were conducted including δ^13^C data from essential AA to identify (a) trophic connections between POM and mesozooplankton (Figure 6) and (b) spatial differences in the δ^13^C signatures of mesozooplankton from different sub‐basins of Baltic Sea (Figure 7). For the outputs of both principal component analyses, see Appendix S1: Table S5. In both PCA analyses, the vector lengths showed that all essential AAs were important for the variations of the first two ordination components. In the first PCA (Figure 6), the first principal component (PC1) explained 47.5%. In PC1, the δ^13^C values in essential AAs of POM samples mainly from the WWs were clustering the most with the mesozooplankton samples. The second principal component (PC2) explained 20.7% of the variation and showed an interanual separation of mesozooplankton between the warmer and the colder summer of 2014 and 2015, respectively. In the second PCA (Figure 7), PC1 explained 54.4% of the variation and identified that δ^13^C signatures in mesozooplankton differed among the five sub‐basins of the Baltic Sea. Along PC1, mesozooplankton data from the EGB were separated from data from the more southern (AB, BB, SGB) and western parts (WB) of the Baltic Sea (Figure 1). PC2 explained 19.8% of the variation and mirrored the same pattern of an interannual separation of mesozooplankton between two contrasting summers as in PCA from Figure 6. Finally, δ^13^C values between the three most informative AAs (Δδ^13^C_Ile‐Leu_ vs. Δδ^13^C_Ile‐Lys_, according to Larsen et al. (2009)) showed that POM samples from this study clustered with the bacterial end‐member values and surprisingly not with microalgae end‐members (Appendix S1: Figure S2).

## 4 DISCUSSION

In context to unpalatable, lipid‐poor, filamentous cyanobacterial blooms, we found that summer mesozooplankton had plasticity only in the lipid levels, but not in proteins. Despite the lipid shortage period in mid‐summer, mesozooplankton species were largely able to cover their lipid demands, while reduced lipids were measured in surface mesozooplankton. By using a multitracer approach (FA trophic markers and δ^13^C patterns in individual FAs and AAs) together with taxonomy data, we revealed that the dietary baseline is complex, with cyanobacteria, mixo‐ and heterotrophic (dino‐) flagellates, and detrital complexes. Due to absent herbivore feeding mode by mesozooplankton, this study emphasized that cyanobacterial FAs and AAs must have been incorporated via feeding on mixo‐ and heterotrophic (dino‐) flagellates and detrital complexes during summer. Night migration of the deep community into the upper waters was linked to the feeding on a (dino‐) flagellate diet that is enriched in essential EPA and DHA to presumably renew their essential FA pool. For the first time, we displayed spatial variations of δ^13^C patterns of essential AAs in surface mesozooplankton across the Baltic Sea. These patterns could distinguish isotopic signatures between sub‐basins of more saline and brackish waters and isotopic signatures of cyanobacteria‐associated food webs.

### 4.1 Plasticity of the lipid and protein pools

Zooming into the lipid pool of the mesozooplankton communities revealed a clear vertical trend of relatively lipid reduced species (2.8%–10.4% of DW) dominated by *T. longicornis* in the upper water layers and lipid‐rich species (25.0%–28.2% of DW) dominated by *Pseudo‐/Paracalanus* spp. in the waters below the halocline. This spatial separation followed the physiological requirements of the species for warmer or more saline waters in the vertical column (Peters et al., 2006, 2013). Overall, such contrast of lipid levels is characteristic for both particular species (Peters et al., 2006, 2013).

The *T. longicornis* mesozooplankton community was dominated mainly by older copepodite stages (unpublished results), and the lipid content was twice as low compared to the only available lipid data of 10.1 ± 0.2% of DW for stage C5‐C6 of *T. longicornis* from waters off southwest Norway (Evjemo et al., 2003). The low lipid levels are more characteristic for *T. longicornis* females (in a range of 5.0%–12.0% of DW; Peters et al., 2013). Namely, the lipid content of zooplankton in the SW made up less than 5% of DW, regardless of whether the dominant species were cladocerans in 2014 or mainly older copepodite stages of *T. longicornis* in 2015 (Tables 1 and 2). In waters off southwest Norway, the diet for copepods consists usually of diatoms and dinoflagellates, while cyanobacteria are absent (Erga & Skjoldal, 1990; Peters, Dutz, & Hagen, 2007). Cyanobacteria typically are assumed to be lipid reduced relative to other microalgae groups (Finkel et al., 2016; Jónasdóttir, 2019), yet filamentous *Nodularia spumigena* and *Aphanizomenon* spp. made up the majority (43%–67%) of the total phytoplankton cell‐carbon at our EGB station during both summers (Eglite et al., 2018). In the Bornholm Basin, low lipid levels and lack of essential FAs in the females of *T. longicornis* together with reduced egg production in summer compared to spring have previously been related to poor feeding conditions (Arendt et al., 2005; Peters et al., 2013). Therefore, these results suggest that especially those mesozooplankton species or copepodite stages are affected by lipid‐poor conditions that are mainly limited to feed in the SW layer above the thermocline, unless it is not a natural effect of an accumulation of stages that are lipid reduced in general. At the same time, the range of lipids (25.0%–28.2% of DW) in the BW community comprising mainly nauplii and older copepodite stages (Appendix S1: Table S4) was comparatively high in comparison with *Pseudo‐/Paracalanus* spp. females (9%–14% of DW, Peters et al., 2006) but were characteristic for *Pseudo‐/Paracalanus* spp. copepodites (26%–32% of DW for stage C5, Peters et al., 2006). Our data also showed relatively high wax ester storages in the BW community (Table 2). In the Bornholm Basin, the wax ester content in C4–C5 stages of *P. acuspes* was around 55% of total lipids during summer, which is lower than the wax ester content of 66%–77% of total lipids at the EGB. This shows that we were mainly collecting wax ester rich copepodite stages that were preparing for overwintering already since May (Peters et al., 2006). Relatively high wax ester content in the EGB also suggested that the *Pseudo‐/Paracalanus* spp.*‐*dominated community experienced good feeding conditions despite the bloom of lipid‐poor cyanobacteria.

The protein content in the UWC mesozooplankton was in a similar range compared to previously measured protein contents in *Temora* spp. from other marine systems without cyanobacteria dominance (Helland, Terjesen, & Berg, 2003; Martynova, Graeve, & Bathmann, 2009). To the best of our knowledge, these are the first data on the protein content of lipid‐rich *Paracalanus* or *Pseudocalanus* species, whose protein content was 50% lower than in the *T. longicornis‐*dominated communities (Table 2). Generally, it confirms that the temperate and boreal mesozooplankton have different energy metabolisms and demands for lipids and proteins (Båmstedt, 1986; Mauchline, 1998). Changes in AA composition (mainly in essential AAs) are usually linked to poor quality food, low lipid levels, and even animals' starvation (Båmstedt & Holt, 1978; Helland, Christian Nejstgaard, et al., 2003; Mayzaud, 1976). In two contrasting summers, the AA composition and content of individual AAs in both dominated communities remained the same despite lipid‐poor cyanobacterial blooms (Table 2 and Appendix S1: Figure S1). No changes in AA pools in summer mesozooplankton (homeostasis between essential and nonessential AAs) despite the presence of lipid‐poor cyanobacterial blooms was also supported by the similarity of mass % THAA values with *T. longicornis* have been feeding on dinoflagellates and diatoms (Guisande et al., 2002). Overall, these results suggest that food webs associated with cyanobacterial blooms contained sufficient amount of proteins and balanced AA composition but can be short of lipids when the feeding grounds are limited to the SW layer and strong seasonal thermocline.

### 4.2 Foraging strategies of key copepod species

High quantities of DHA and 18:4(n‐3) together with phytoplankton taxonomy data from Eglite et al. (2018) corroborate the preferential feeding of the mesozooplankton communities on flagellates in the EGB during summer. Despite the dominance of cyanobacteria, *Dinophyceae* (dinoflagellates, namely mixotrophic *Dinophysis norvegica*, heterotrophic *Protoperidinium* spp.) and *Prymnesiophyceae* (flagellates, namely mixotrophic *Prymnesium* spp.) still made up for relatively high 24%–37% of the total microplankton cell‐carbon biomass at our station during both years (Eglite et al., 2018; Wasmund, Dutz, Pollehne, Siegel, & Zettler, 2015, 2016). Studies from the Bornholm Basin and off central Norway showed that heterotrophic dinoflagellates and small flagellates are important food sources for *T. longicornis* especially in summer (Dutz, Mohrholz, & van Beusekom, 2010; Dutz, Van Beusekom, & Hinrichs, 2012; Evjemo, Tokle, Vadstein, & Olsen, 2008). Furthermore, such preferential grazing on mixo‐ and heterotrophic (dino‐) flagellates can explain the predominantly omni‐ and carnivorous feeding modes due to TPs > 2.5 (Table 2).

We also observed relatively high contents of EPA (Figure 2a,b). Typically, EPA together with 16:1(n‐7) is associated with a diatom‐based diet (Graeve et al., 1994; Volkman et al., 1989). In the Baltic Sea, the biomass of diatoms usually declines after spring due to the seasonal phytoplankton succession (Wasmund, Nausch, & Matthäus, 1998). Consequently, diatoms' biomass was limited (less than 7% of the total microplankton cell‐carbon biomass) or was absent at our station (Eglite et al., 2018; Wasmund, Dutz, Pollehne, Siegel, & Zettler, 2015, 2016). Apart from diatoms, large quantities of a combination of EPA and DHA have earlier been found also in (dino‐) flagellates (Ahlgren et al., 1992; Mansour, Volkman, Jackson, & Blackburn, 1999; Reitan, Rainuzzo, & Olsen, 1994). Thus, due to low biomass or absence of diatoms during both summers, in combination with a relatively low content of diatom FA trophic marker 16:1(n‐7), we conclude that EPA (Figure 2a,b) must have originated from other phytoplankton groups, such as flagellates and dinoflagellates.

The occurrence of FAs 18:2(n‐6) and 18:3(n‐3) in both mesozooplankton communities supported the assumption of incorporation of cyanobacterial FAs (Figure 2a,b). The incorporation of cyanobacterial food sources in the same UWC mesozooplankton was also proposed by Eglite et al. (2018), due to comparatively low δ^15^N‐Phe values (a proxy for N sources) that is a result of diazotroph Phe entering (an AA synthesized from ammonium that originated from N_2_ fixation) in the animals' biomass. At the same time, the absence of a herbivory signal excludes direct feeding on photoautotrophs and a direct uptake of cyanobacterial FA trophic markers from the dominating cyanobacteria *N. spumigena* in both summers (Table 2, Eglite et al., 2018). In the Baltic Sea, the *N. spumigena* filaments are the substrate for high bacterial productivity that attracts microzooplankton, especially in the late bloom phase, and improves the quality of cyanobacterial food source for copepods (Hoppe, 1981). Recently, Loick‐Wilde et al. (2019) showed an increase in surface mesozooplankton mean TPs, associated with the decaying of cyanobacterial blooms in the central Baltic Sea, which was also accompanied by increasing microplankton diversity. Consequently, the UWC community must have received cyanobacterial FAs via the microbial food web that degrade aging *N. spumigena* blooms (Hoppe, 1981).

The relatively high quantities of cyanobacterial FA markers in the deep mesozooplankton are ambiguous (Figure 2a), because mesozooplankton in the BW were characterized by isotopically enriched δ^15^N‐Phe values (Eglite et al., 2018). This means isolation of the BW community from the diazotrophic N sources in the SWs (that usually have depleted δ^15^N values), even though in both years 60% of the total phytoplankton biomass in the SW consisted of unpalatable cyanobacteria filaments (Eglite et al., 2018). In contrast to AAs (Loick‐Wilde et al., 2018, 2019), it remains unclear to which degree cyanobacterial FAs degrade during decaying cyanobacteria blooms in the SW. Still, a relatively high content of cyanobacterial FAs in the deep community suggests that cyanobacteria must be part of sinking POM or detritus and reached the BW. Decaying cyanobacterial aggregates of *Aphanizomenon* spp. and *N. spumigena* can sink out rapidly (Ploug, 2008), and the presence of cyanobacteria on sediments of the Baltic Sea indicated a direct sinking of these aggregates (Suikkanen, Kaartokallio, Hällfors, Huttunen, & Laamanen, 2010), that even can stimulate benthic production (Karlson et al., 2015). Feeding on sinking matter that might be decayed by the BW community was supported by a very high proportion of 18:1(n‐9) trophic marker for detritus (Kattner & Krause, 1989; Figure 2a) and a significant linear relationship with the microbial degradation index ΣV (Figure 5a). Nevertheless, the 18:1(n‐9) may also be synthesized de novo via desaturation of 18:0 (Kattner & Hagen, 1995), but it is rather unlikely as long as enough dietary resources of 18:1(n‐9) are available (Dalsgaard et al., 2003). According to the mass‐balance rule, the FAs that derived from elongation and/or desaturation processes during de novo synthesis are expected to produce enriched δ^13^C values in the substrate pool of the consumers' lipids (Peterson & Fry, 1987). In our case, the isotopic differences were inconsistent between 18:0 (substrate) and 18:1(n‐9) (product), thus suggesting that 18:1(n‐9) at least partly was incorporated from dietary FAs.

Our study supported earlier observations of diurnal migration by deep *Pseudo‐/Paracalanus* spp. community during the nighttime in the Bornholm Sea, namely by the younger stages (Peters et al., 2006). At our station, the diurnal changes of 18:1(n‐9) content and wax ester storage lipid variations were suggested to be originated from migration of *Pseudo‐/Paracalanus* spp. (Appendix S1: Table S1, Peters et al., 2006), since *T. longicornis* is low in these compounds (Peters et al., 2013). It certainly raises the question of why the younger stages of *Pseudo‐/Paracalanus* spp. migrate from the BW into the UWC at night. Such behavior could be linked to the zooplankton needs for high‐quality food sources and presumably essential EPA and DHA, which were found in high quantities in the UWC mesozooplankton (Figures 4b and 5). Supporting this, (dino‐) flagellates have earlier been suggested to be a part of the diet for *P. acuspes* in the Bornholm Basin during summer (Peters et al., 2006), and *Pseudo‐/Paracalanus* spp. was also found in the WW layer at the EGB (Figure 5). Accordingly, we suggest on a simultaneous feeding in the subthermocline waters (~10–65 m) by both key copepod species on (dino‐) flagellates that are rich in essential EPA and DHA (Ahlgren et al., 1992; Reitan et al., 1994). Yet, during warmer summers, this might increase a competition between both species for the same preferred diet. We provided evidence that only during the hot summer of 2014 (SST > 19.0°C) *T. longicornis* individuals and the characteristic wax esters of *Pseudo‐/Paracalanus* spp. were absent in mesozooplankton from the SW (>10 m, Table 1), while the biomass peak of *T. longicornis* and *Pseudo‐/Paracalanus* spp. was found in the colder subthermocline waters (~10–65 m, ~4°C; Table 1), under conditions that are more favorable to both species (Dutz et al., 2010; Peters et al., 2013). Moreover, in a complementary study by Eglite et al. (2018) it was shown that the total phytoplankton cell‐C concentrations decreased with depth that could not only be a result from light limitation but additionally from feeding pressure by abundant mesozooplankton.

### 4.3 Mesozooplankton metabolism and dietary sources according to δ^13^C patterns of fatty acids and amino acids

Similar δ^13^C patterns of nonessential AAs in mesozooplankton revealed that both communities have alike AA metabolism that resulted in the same definite isotopic patterns (Figure 4a,b). In contrast, differences in the δ^13^C values of their nonessential FAs could be linked to community‐specific C isotope fractionation during lipid metabolism (Figure 3a,b). Contrasting lipid metabolism strategies between temperate and boreal communities became visible via a significant positive correlation of wax ester content with δ^13^C values of the FA 18:1(n‐9) (Figure 5b). The FA 18:1(n‐9) accumulates in high amounts in the wax ester fraction of zooplankton and is involved in the wax ester biosynthesis, thus supporting the long‐term energy deposits (Falk‐Petersen et al., 2000; Lee, Hagen, & Kattner, 2006). In the EGB, continuous enrichment of δ^13^C values in 18:1(n‐9) suggested that this FA is used as a precursor in lipid metabolism and is linked to the wax ester synthesis in the *Pseudo‐/Paracalanus* spp.‐dominated community (Figure 5b).

In this study, we lack data of δ^13^C values in individual FAs of POM samples. However, we still found that in mesozooplankton δ^13^C values of essential FAs with 18C are significantly different from essential δ^13^C‐EPA and δ^13^C‐DHA values, while both EPA and DHA have similar δ^13^C values to bulk δ^13^C in POM. Such differences have mainly been attributed to FA‐specific trophic fractionation of ^13^C isotope that depends on the individual FA structure (Bec et al., 2011; Budge, Wang, Hollmen, & Wooller, 2011). Similar δ^13^C values of dinoflagellate‐FA trophic markers of EPA and DHA in mesozooplankton with bulk δ^13^C values in POM additionally supported FA trophic marker data and that both essential FAs must be entering in the mesozooplankton via feeding on a (dino‐) flagellate source. In contrast, the δ^13^C values of most essential AAs in mesozooplankton were similar to bulk δ^13^C values in POM, except for Thr (Figure 4a,b). Since trophic fractionation in essential AAs was found to be minor, δ^13^C differences in Thr values probably were a result of an imbalance between mesozooplankton and its POM diet (McMahon et al., 2010). In our study, overall δ^13^C patterns of essential AAs between field POM and mesozooplankton samples were clustered close to each other, especially with the POM from the WWs (Figure 6). This suggests that δ^13^C values of individual AAs in POM at least partly represent integrated AA sources of associated mesozooplankton, while mesozooplankton prefers one food source over others in the POM mixture. In the EGB δ^13^C values of POM, the three most informative AAs (Larsen et al., 2009) resembled bacteria, and surprisingly not the microalgae end‐members (Appendix S1: Figure S2). Thus, the isotopic signatures of POM seem to be altered by microbial reworking that can cause a shift in δ^13^C patterns (Larsen et al., 2013) and information of microalgae AA sources might be lost. An early study from the EGB showed high bacterial diversity associated with the POM particles especially in summer (Rieck, Herlemann, Jürgens, & Grossart, 2015). Additionally, in a complementary studies by Loick‐Wilde et al. (2018) concentrations of THAA‐N in the PON fraction were less than 30 mole %, which indicates a highly degraded state of POM at EGB station (Cowie & Hedges, 1994). Nevertheless, similar δ^13^C essential AA patterns between POM that resembled bacteria (Appendix S1: Figure S2) and mesozooplankton (Figure 6) supported that the key copepod species must be feeding also on detrital complexes at the EGB station (Peters et al., 2013; Poulet, 1976). Overall these results of essential FA and AA δ^13^C values together with FA trophic markers underline that in summer the ecosystem of the EGB is complex, comprising of cyanobacteria, mixo‐ and heterotrophic (dino‐) flagellates, and detrital complexes that contributed to the planktonic food web. We suggest that more POM data together with taxon‐specific δ^13^C data of AAs and FAs of microplankton end‐members (e.g., Nostocophyceae and Dinophyceae) and detritus that are isolated from a particular environment need to be analyzed. These results can be applied for advanced source diagnostic analysis (e.g., δ^13^C essential AA fingerprinting, Larsen et al., 2013) for characterizing a complex dietary baseline that is associated with the decaying cyanobacterial blooms in the Baltic Sea (Loick‐Wilde et al., 2019; Wasmund, 1997).

### 4.4 δ^13^C patterns of essential amino acids for basin‐specific isoscapes

The patterns of δ^13^C essential AAs in mesozooplankton clearly distinguished the planktonic food web at the EGB station with the abundant cyanobacteria biomass from the other sub‐basins where the cyanobacteria biomass was reduced, for example, the WE and the AB. (Loick‐Wilde et al., 2019; Wasmund, Dutz, Pollehne, Siegel, & Zettler, 2015, 2016). Presumably, these changes in δ^13^C values of essential AAs in surface mesozooplankton are evidence that the cyanobacteria‐based food web is isotopically different from the diatom‐ or (dino‐) flagellate‐based food webs in the southwest of the Baltic Sea (Loick‐Wilde et al., 2019; Wasmund et al., 2015, 2016). The δ^13^C‐AA patterns in the Baltic Sea could also distinguish the mesozooplankton communities along the surface salinity gradient from more saline waters in the southwest and more brackish waters at the EGB (Leppäranta & Myrberg, 2009). Environmental parameters such as salinity seem to regulate the δ^13^C‐AA patterns in surface mesozooplankton across the sub‐basins in the Baltic Sea (Pronin, Panettieri, Torn, & Rumpel, 2019). Moreover, during the hot and the cold summer, we observed interannual variability of δ^13^C‐AA patterns in mesozooplankton (driven by PC2, Figure 7, only available data for the EGB station) that we primarily link to changes in environmental conditions, such as contrasting SST (Eglite et al., 2018). Thus, two contrasting summers showed that changes in environmental conditions at the regional scale can also alter the δ^13^C patterns of planktonic food webs, and thus, environmental conditions need to be considered in order to eliminate any uncertainties in the migration studies. Nevertheless, in our study, the PCA of only a few mesozooplankton values in some sub‐basins was scattered, and more mesozooplankton data are needed for construction of highly detailed ecosystem‐wide δ^13^C isoscapes in the Baltic Sea. Potentially, these mesozooplankton δ^13^C isoscapes can be used to identify the commercial fish feeding trails, for example, for herring and sprat in the Baltic Sea (Möllmann & Köster, 2002; Torniainen et al., 2017) from more saline waters in the southwest to the more brackish waters in the EGB (Leppäranta & Myrberg, 2009) and in cyanobacteria‐associated food webs.

## CONFLICT OF INTERESTS

All authors declare no conflict of interest.

## AUTHOR CONTRIBUTIONS

E.E and N.L.W conceptualize this study and collected samples. M.G. conducted the lipid and fatty acid analyses at the Alfred Wegener Institute. E.E derivatized and prepared all samples for further amino acid analysis. E.E and D.W performed the protein and amino acid analyses at the Leibniz Institute for Baltic Sea Research. I.L. was responsible about the bulk stable isotope analyses. E.E, M.G., J.D., D.S.B., N.L.W evaluated the data. E.E produced tables, figures and wrote the manuscript. All authors contributed to the preparation of the manuscript.

## Data Availability

Raw data of biochemical and stable isotopes analyses are archived on Dryad (https://doi.org/10.5061/dryad.tq63tp2). Part of the data available in the Appendix S1.
