# Supplementary material for: Metabolism and foraging strategies of mid‐latitude mesozooplankton during cyanobacterial blooms as revealed by fatty acids, amino acids, and their stable carbon isotopes
Source: Ecol Evol. 2019 Aug 16;9(17):9916–34. doi: 10.1002/ece3.5533 (PMC6745671; doi:10.1002/ece3.5533)
Supplement: Supplementary file 1 [file ECE3-9-9916-s001.docx]

**APPENDIX S1**

**Supportive online material for:** **Metabolism and foraging strategies of mid‐latitude mesozooplankton during cyanobacterial blooms as revealed by fatty acids, amino acids, and their stable carbon isotopes**

**Table S1.** Diurnal wax ester content changes **(**% of total lipids) and stable carbon isotope values (δ^13^C. ‰ vs PDB) in the non-essential fatty acid 18:1 (n-9) in two mesozooplankton size fractions from specific depths and sampling times at the Eastern Gotland Basin in July 2014 and 2015.

|  |  | | **2014** | | | | |  |
| --- | --- | --- | --- | --- | --- | --- | --- | --- |
|  |  | | **Day-time** | | | **Night-time** | | |
| Depth | Fraction | | Sampling  time (UTC) | wax esters | δ^13^C of 18:1 (n-9) | Sampling  time (UTC) | wax esters | δ^13^C of 18:1 (n-9) |
| 0-10m | 100-300µm | | 13:58 | 0.00 | -38.4 | 23:42 | 0.0 | -39.1 |
| 0-10m | 300µm | | 13:58 | n/s | n/s | 23:42 | 0.0 | -36.4 |
| 10-60m | 100-300µm | | 13:25 | 14.9 | -34.6 | 23:15 | 55.3 | -33.1 |
| 10-60m | 300µm | | 13:25 | 14.7 | -34.1 | 23:15 | 54.2 | -23.1 |
| 60-125m | 100-300µm | | 12:16 | 69.8 | -30.3 | 22:29 | 75.9 | -32.3 |
| 60-125m | 300µm | | 12:16 | 65.8 | -30.1 | 22:29 | 66.8 | -30.9 |
| mean ± SD | |  |  | 33.0±32.4 | -33.5±3.5 |  | 42.0±33.5 | -32.3±5.5 |
|  |  | |  |  |  |  |  |  |
|  |  | |  |  |  |  |  |  |
|  |  | | **2015** | | | | |  |
|  |  | | **Day-time** | | | **Night-time** | |  |
| Depth | Fraction | | Sampling  time (UTC) | wax esters | δ^13^C of 18:1 (n-9) | Sampling  time (UTC) | wax esters | δ^13^C of 18:1 (n-9) |
| 0-25m | 100-300µm | | 09:20 | 43.0 | -28.6 | 23:10 | 0.8 | -37.9 |
| 0-25m | 100-300µm | | 19:21 | 1.6 | -37.7 | 01:25 | 5.3 | -35.1 |
| 0-25m | 300µm | | 09:20 | n/s | n/s | 23:10 | 3.1 | -38.1 |
| 0-25m | 300µm | | 19:21 | n/s | -37.3 | 01:25 | 0.0 | -36.5 |
| 25-65m | 100-300µm | | 08:30 | 29.6 | -30.1 | 22:10 | 58.0 | -30.6 |
| 25-65m | 100-300µm | | 18:15 | 38.2 | -29.9 | 02:05 | 47.2 | -25.8 |
| 25-65m | 300µm | | 08:30 | 0.0 | -40.2 | 22:10 | 61.3 | -28.7 |
| 25-65m | 300µm | | 18:15 | 25.3 | -29.4 | 02:05 | 14.6 | -30.0 |
| mean ± SD | |  |  | 19.6±18.8 | -33.3±4.4 |  | 23.8±26.9 | -32.8±4.6 |

n/s – not sufficient sample material

**Table S2.** Total lipids (% of dry weight), wax esters (% of total lipids), composition of fatty acids and fatty alcohols (% of total fatty acids and alcohols, respectively) in two mesozooplankton size-fractions from three water bodies (surface water (SW), winter water (WW) and bottom water (BW)) at the Eastern Gotland Basin in July of 2014 and 2015.

|  | 2014 | 2015 |  | 2014 | 2015 |  | 2014 |
| --- | --- | --- | --- | --- | --- | --- | --- |
|  | SW | SW |  | WW | WW |  | BW |
|  | 0-10m | 0-25m |  | 10-60m | 25-65m |  | 65-125m |
| Size  fraction (μm) | 100-300 | 100-300 |  | 100-300 | 100-300 |  | 100-300 |
|  | *(n*=2) | (*n*=4) |  | (*n*=2) | (*n*=4) |  | (*n*=2) |
| **Total lipids** | 3.8 ± 0.6 | 4.7 ± 2.0 |  | 6.9 ± 3.7 | 9.2 ± 2.8 |  | 25.0 ± 0.8 |
| **Wax esters** | - | 12.7 ± 20.3 |  | 35.1 ± 28.5 | 43.2 ± 12.2 |  | 72.8 ± 4.3 |
| **Fatty acid** |  |  |  |  |  |  |  |
| 14:0 | 7.5 ± 3.8 | 3.9 ± 1.1 |  | 1.9 ± 0.7 | 2.3 ± 0.8 |  | *tr* |
| 15:0 | *tr* | *tr* |  | *tr* | *tr* |  | *-* |
| 16:0 | 19.9 ± 0.7 | 18.2 ± 3.8 |  | 13.6 ± 5.3 | 12.2 ± 3.4 |  | 5.2 ± 0.1 |
| 17:0 | *tr* | *tr* |  | 0.6 ± 0.8 | *tr* |  | - |
| 18:0 | 4.7 ± 0.6 | 3.3 ± 0.9 |  | 2.5 ± 1.4 | 1.8 ± 0.5 |  | *tr* |
| 20:0 | - | *tr* |  | *-* | 0.5 ± 0.7 |  | 1.5 ± 0.1 |
| ∑SFAs | 32.7 ± 5.3 | 26.4 ± 5.8 |  | 18.8 ± 8.6 | 17.3 ± 5.0 |  | 7.9 ± 0.1 |
| 16:1(n-9) | *tr* | - |  | - | - |  | *tr* |
| 16:1(n-7) | 6.8 ± 4.0 | 1.9 ± 0.8 |  | 2.4 ± 1.1 | 3.5 ± 1.2 |  | 4.2 ± 0.4 |
| 18:1(n-9) | 4.6 ± 1.4 | 7.8 ± ***8.7*** |  | 18.2 ± ***13.6*** | 21.8 ± ***7.9*** |  | 43.1 ± ***2.4*** |
| 18:1(n-7) | 3.4 ± 0.9 | 2.2 ± 0.4 |  | 1.6 ± 0.3 | 1.7 ± 0.2 |  | 0.6 ± 0.8 |
| 18:1(n-5) | - | *tr* |  | *tr* | *tr* |  | *tr* |
| 20:1(n-9) | 0.5 ± 0.8 | 1.1 ± 0.2 |  | *tr* | 1.1 ± 0.5 |  | - |
| 24:1(n-9) | 1.5 ± 0.7 | 0.7 ± 1.4 |  | 1.7 ± 0.6 | - |  | *tr* |
| ∑MUFAs | 17.4 ± 5.4 | 13.9 ± 8.9 |  | 24.4 ± 13.0 | 28.8 ± 9.2 |  | 48.8 ± 0.7 |
| 16:2(n-4) | 1.0 ± 0.4 | *tr* |  | *-* | *tr* |  | *-* |
| 16:3(n-4) | - | 0.7 ± 0.5 |  | *-* | *tr* |  | *-* |
| 16:4(n-1) | - | - |  | *-* | *-* |  | *-* |
| 18:2(n-6) | 3.2 ± 0.3 | 3.0 ± 1.2 |  | 5.3 ± 2.9 | 4.9 ± 0.8 |  | 11.6 ± 0.3 |
| 18:3(n-3) | 4.1 ± 0.4 | 4.0 ± 1.4 |  | 6.7 ± 3.7 | 5.5 ± 1.4 |  | 7.4 ± 0.3 |
| 18:4(n-3) | 3.1 ± 0.2 | 3.7 ± 1.2 |  | 5.0 ± 2.4 | 5.0 ± 0.8 |  | 6.2 ± 0.1 |
| 20:3(n-6) | *tr* | *tr* |  | - | - |  | - |
| 20:4(n-6) | 1.3 ± 0.1 | *tr* |  | 1.2 ± 0.4 | *tr* |  | - |
| 20:3(n-3) | *tr* | *tr* |  | *tr* | *tr* |  | *-* |
| 20:4(n-3) | *tr* | 0.6 ± 0.5 |  | 1.4 ± 0.8 | 1.5 ± 0.3 |  | 1.9 ± 0.0 |
| 20:5(n-3) EPA | 14.9 ± 1.2 | 14.1 ± 1.6 |  | 13.5 ± 2.8 | 12.3 ± 1.2 |  | 8.6 ± 0.4 |
| 22:5(n-3) | *tr* | *tr* |  | *-* | *tr* |  | - |
| 22:6(n-3) DHA | 20.7 ± *7.8* | 29.6 ± *5.4* |  | 22.3 ± *10.5* | 21.6 ± 3.7 |  | 7.5 ± 0.5 |
| ∑PUFAs | 49.0 ± *9.9* | 57.7 ± 3.5 |  | 55.8 ± 3.8 | 52.2 ± 4.2 |  | 43.2 ± 0.8 |
| **Fatty alcohol** |  |  |  |  |  |  |  |
| 14:0 | - | 57.0 ± 30.0 |  | 30.2 ± 1.2 | 35.2 ± 1.4 |  | 31.1 ± 0.1 |
| 16:0 | - | 57.3 ± 10.8 |  | 69.8 ± 1.2 | 64.8 ± 1.4 |  | 68.9 ± 0.1 |

**Table S2.** Continued. Size fraction ≥300 μm

|  | 2014 | 2015 |  | 2014 | 2015 |  | 2014 |
| --- | --- | --- | --- | --- | --- | --- | --- |
|  | SW | SW |  | WW | WW |  | BW |
|  | 0-10m | 0 - 25m |  | 10-60m | 25-65m |  | 65-125m |
| Size  fraction (μm) | ≥300 | ≥300 |  | ≥300 | ≥300 |  | ≥300 |
|  | (*n*=1) | (*n*=3) |  | (*n*=2) | (*n*=3) |  | (*n*=2) |
| **Total lipids** | 3.9 | 2.8 ± 0.8 |  | 9.7 ± 6.0 | 10.4 ± 10.3 |  | 28.2 ± 9.5 |
| **Wax esters** | - | 1.0 ± 1.8 |  | 34.3 ± 28.1 | 25.3 ± 26.2 |  | 66.3 ± 0.7 |
| **Fatty acid** |  |  |  |  |  |  |  |
| 14:0 | 3.9 | 4.1 ± 0.5 |  | 1.8 ± 1.1 | 3.2 ± 1.6 |  | *tr* |
| 15:0 | *tr* | *tr* |  | *tr* | *tr* |  | - |
| 16:0 | 19.2 | 20.4 ± 0.8 |  | 13.3 ± 6.9 | 15.5 ± 6.3 |  | 6.1 ± 0.6 |
| 17:0 | - | 0.7 ± 0.6 |  | *tr* | tr |  | - |
| 18:0 | 4.0 | 3.8 ± 0.4 |  | 2.0 ± 1.1 | 2.5 ± 1.4 |  | *tr* |
| 20:0 | - | - |  | - | *tr* |  | 1.2 ± 0.1 |
| ∑SFAs | 27.5 | 29.8 ± 1.9 |  | 17.4 ± 9.6 | 22.3 ± 9.8 |  | 8.5 ± 0.7 |
| 16:1(n-9) | - | - |  | - | - |  | *tr* |
| 16:1(n-7) | 2.8 | *tr* |  | 2.2 ± 0.8 | 3.3 ± 2.2 |  | 3.4 ± 0.2 |
| 18:1(n-9) | 3.0 | 2.6 ± 0.8 |  | 20.0 ± ***14.5*** | 14.7 ± ***14.9*** |  | 41.0 ± 1.0 |
| 18:1(n-7) | 2.5 | 2.6 ± 0.0 |  | 1.7 ± 0.4 | 2.0 ± 0.4 |  | 1.3 ± 0.1 |
| 18:1(n-5) | *-* | *-* |  | *tr* | *tr* |  | - |
| 20:1(n-9) | *tr* | *tr* |  | *tr* | 1.3 ± 0.7 |  | - |
| 24:1(n-9) | 2.7 | 2.2 ± 2.0 |  | 1.8 ± 0.8 | - |  | *tr* |
| ∑MUFAs | 11.9 | 9.3 ± 2.1 |  | 26.6 ± 14.3 | 21.8 ± 17.6 |  | 46.2 ± 0.5 |
| 16:2(n-4) | 1.2 | 0.4 ± 0.7 |  | 0.7 ± 0.4 | 0.8 ± 0.3 |  | - |
| 16:3(n-4) | - | - |  | - | - |  | - |
| 16:4(n-1) | - | - |  | - | - |  | - |
| 18:2(n-6) | 2.7 | 2.1 ± 0.2 |  | 7.3 ± 4.4 | 3.5 ± 1.4 |  | 14.7 ± 0.1 |
| 18:3(n-3) | 3.1 | 2.6 ± 0.3 |  | 5.9 ± 2.7 | 3.5 ± 0.1 |  | 7.7 ± 0.1 |
| 18:4(n-3) | 2.5 | 2.5 ± 0.3 |  | 4.7 ± 1.9 | 3.8 ± 0.4 |  | 6.6 ± 0.1 |
| 20:3(n-6) | - | - |  | 0.6 ± 0.8 | - |  | - |
| 20:4(n-6) | 1.2 | *tr* |  | 0.9 ± 0.4 | *tr* |  | - |
| 20:3(n-3) | *tr* | *tr* |  | *tr* | *tr* |  | - |
| 20:4(n-3) | *tr* | *tr* |  | 1.4 ± 0.8 | 0.9 ± 0.8 |  | 1.9 ± 0.1 |
| 20:5(n-3) EPA | 17.5 | 16.1 ± 0.4 |  | 13.6 ± 3.2 | 14.0 ± 2.3 |  | 8.1 ± 0.1 |
| 22:5(n-3) | *tr* | *tr* |  | *tr* | *tr* |  | - |
| 22:6(n-3) DHA | 28.8 | 33.5 ± 4.0 |  | 19.2 ± 9.0 | 26.4 ± 8.0 |  | 6.2 ± 0.0 |
| ∑PUFAs | 58.7 | 58.9 ± 3.9 |  | 54.8 ± 4.4 | 54.0 ± 8.3 |  | 45.3 ± 0.2 |
| **Fatty alcohol** |  |  |  |  |  |  |  |
| 14:0 | - | 43.8 |  | 30.4 ± 0.7 | 37.3 ± 0.2 |  | 30.4 ± 0.1 |
| 16:0 | - | 56.2 |  | 69.6 ± 0.7 | 62.7 ± 0.2 |  | 69.6 ± 0.1 |

Notes: *n* – number of samples within 24 hours. *tr –* trace FAs below the <1% of total FAs. Values are given as a mean over the diel sampling ± SD.

**Table S3.** Total proteins (% of dry weight) and amino acid composition (% of total proteins) in two mesozooplankton size-fractions from three water bodies (surface water (SW), winter water (WW) and bottom water (BW)) at the Eastern Gotland Basin in July 2014 and 2015.

|  | 2014 | 2015 |  | 2014 |  | 2015 |  | 2014 |
| --- | --- | --- | --- | --- | --- | --- | --- | --- |
|  | SW | SW |  | WW |  | WW |  | BW |
|  | 0-10m | 0 - 25m |  | 10-60m |  | 25-65m |  | 65-125m |
| Size  fraction (μm) | 100-300 | 100-300 |  | 100-300 |  | 100-300 |  | 100-300 |
|  | (*n*=1 / 2) | (*n*=4) |  | (*n*=2) |  | (*n*=4) |  | (*n*=2) |
| **Total proteins** | 43.4 ± 1.5 | 38.4 ± 8.5 |  | 42.5 ± 3.9 |  | 30.6 ± 4.7 |  | 29.6 ± 0.5 |
| **Amino acids (AAs)** |  |  |  |  |  |  |  |  |
| Aspartic acid* | 10.8 | 11.3 ± 2.9 |  | 15.7 ± 1.0 |  | 11.3 ± 1.5 |  | 11.8 ± 0.2 |
| Alanine | 8.0 | 12.8 ± 4.0 |  | 9.6 ± 3.2 |  | 11.0 ± 1.7 |  | 8.4 ± 0.0 |
| Glycine | 5.8 | 7.6 ± 2.3 |  | 5.3 ± 2.6 |  | 6.4 ± 0.7 |  | 5.4 ± 0.2 |
| Glutamic acid** | 14.3 | 17.4 ± 1.6 |  | 21.1 ± 1.0 |  | 17.5 ± 1.1 |  | 16.7 ± 1.0 |
| Proline | 4.9 | 6.0 ± 2.3 |  | 6.8 ± 1.2 |  | 5.1 ± 0.3 |  | 4.8 ± 0.0 |
| Serine | 5.3 | 2.0 ± 1.2 |  | 5.4 ± 0.9 |  | 3.0 ± 1.4 |  | 4.3 ± 0.3 |
| ∑Non-essential AAs | 49.1 | 57.1 ± 5.3 |  | 50.1 ± 11.7 |  | 54.2 ± 0.6 |  | 51.4 ± 0.6 |
| Leucine | 9.0 | 9.8 ± 0.2 |  | 11.5 ± 1.7 |  | 9.3 ± 0.4 |  | 8.8 ± 0.3 |
| Lysine | 10.1 | 11.8 ± 0.9 |  | 12.5 ± 1.0 |  | 11.9 ± 0.5 |  | 12.9 ± 0.2 |
| Isoleucine | 5.5 | 4.6 ± 2.3 |  | 6.6 ± 1.2 |  | 5.4 ± 0.7 |  | 5.0 ± 0.1 |
| Phenylalanine | 5.3 | 5.0 ± 0.4 |  | 6.5 ± 0.5 |  | 4.6 ± 0.2 |  | 4.1 ± 0.0 |
| Threonine | 5.7 | 4.0 ± 0.8 |  | 6.9 ± 1.1 |  | 5.0 ± 0.3 |  | 5.3 ± 0.2 |
| Tyrosine | 7.9 | *tr* |  | 2.7 ± 3.1 |  | 1.1 ± 1.0 |  | 5.5 ± 0.1 |
| Valine | 6.7 | 6.7 ± 3.0 |  | 8.2 ± 1.9 |  | 7.9 ± 1.1 |  | 6.8 ± 0.0 |
| ∑Essential AAs | 50.3 | 42.6 ± 5.2 |  | 45.2 ± 1.1 |  | 49.4 ± 11.2 |  | 48.6 ± 0.7 |

**Table S3**. Continued. Size fraction *≥*300 μm.

|  | 2014 | 2015 |  | 2014 | 201~~5~~ |  | 2014 |
| --- | --- | --- | --- | --- | --- | --- | --- |
|  | SW | SW |  | WW | WW |  | BW |
|  | 0-10m | 0 - 25m |  | 10-60m | 25-65m |  | 65-125m |
| Size  fraction (μm) | ≥300 | ≥300 |  | ≥300 | ≥300 |  | ≥300 |
|  | (*n*=1 / 2) | (*n*=4) |  | (*n*=2) | (*n*=4) |  | (*n*=2) |
| **Total proteins** | 47.4 ± 0.8 | 49.6 ± 9.3 |  | 47.6 ± 8.4 | 41.4 ± 1.6 |  | 25.1 ± 1.6 |
| **Amino acids** |  |  |  |  |  |  |  |
| Aspartic acid* | 10.6 | 10.4 ± 2.5 |  | 12.9 ± 0.6 | 12.0 ± 0.1 |  | 12.4 ± 0.0 |
| Alanine | 7.9 | 10.0 ± 0.4 |  | 7.5 ± 1.5 | 10.3 ± 1.1 |  | 6.9 ± 0.0 |
| Glycine | 6.1 | 6.0 ± 0.2 |  | 5.2 ± 0.8 | 6.2 ± 0.6 |  | 4.9 ± 0.1 |
| Glutamic acid* | 13.5 | 18.7 ± 1.1 |  | 17.5 ± 0.8 | 17.6 ± 0.4 |  | 17.8 ± 0.6 |
| Proline | 4.1 | 4.9 ± 0.1 |  | 4.7 ± 0.1 | 4.8 ± 0.1 |  | 4.7 ± 0.1 |
| Serine | 5.1 | 2.5 ± 0.9 |  | 4.2 ± 0.0 | 3.0 ± 1.9 |  | 4.2 ± 0.2 |
| ∑Non-essential AAs | 47.4 | 52.5 ± 1.1 |  | 52.0 ± 0.8 | 53.9 ± 0.6 |  | 50.9 ± 0.7 |
| Leucine | 8.9 | 9.6 ± 0.1 |  | 9.3 ± 0.5 | 9.3 ± 0.6 |  | 9.2 ± 0.0 |
| Lysine | 11.4 | 12.0 ± 0.6 |  | 12.9 ± 1.0 | 11.3 ± 0.5 |  | 13.1 ± 0.1 |
| Isoleucine | 6.2 | 5.7 ± 0.3 |  | 5.4 ± 0.4 | 5.4 ± 0.8 |  | 4.9 ± 0.0 |
| Phenylalanine | 5.2 | 5.0 ± 0.1 |  | 4.7 ± 1.2 | 4.6 ± 0.3 |  | 4.5 ± 0.0 |
| Threonine | 6.2 | 4.8 ± 0.5 |  | 5.7 ± 0.4 | 4.8 ± 0.7 |  | 5.0 ± 0.0 |
| Tyrosine | 5.1 | 0.8 ± 0.6 |  | 2.4 ± 1.0 | 1.6 ± 1.6 |  | 6.1 ± 0.5 |
| Valine | 6.9 | 7.5 ± 0.4 |  | 6.9 ± 1.0 | 7.8 ± 1.1 |  | 6.1 ± 0.0 |
| ∑Essential AAs | 49.9 | 45.4 ± 1.6 |  | 44.9 ± 1.2 | 47.2 ± 1.0 |  | 48.9 ± 0.5 |

Notes: *n* - number of samples within 24 hours. Values are given as mean of *n* over diel sampling ± SD; *- sum of Asn+Asp **- sum of Gln+Glu.

**Table S4.** Abundance of *Pseudo-* and *Paracalanus* spp*.* stages at different depths in July 2014 and 2015 in the Eastern Gotland Basin. Abbreviations of water bodies: surface water (SW), winter water (WW), bottom water (BW). See text for more details.

Mesozooplankton abundance (ind m^-3^)

|  |  |  | 2014 |  |  |  | 2015 |  |
| --- | --- | --- | --- | --- | --- | --- | --- | --- |
|  |  |  |  |  |  |  |  |  |
| **Copepods** |  | 0-10m | 10-60m | 60-125m |  | 0-25m | 25-65m | 65-110m |
|  |  | (SW) | (WW) | (BW) |  | (SW) | (WW) | (BW) |
|  |  |  |  |  |  |  |  |  |
| *Pseudo/Paracalanus spp.* | | 157 | 1791 | 2834 |  | 240 | 1713 | 2467 |
| Division in stages: |  |  |  |  |  |  |  |  |
| Nauplii(N1→N6) |  | - | 353 | 28 |  | 200 | 138 | - |
| Copepodites (C1→C3) | | 90 | 1211 | 842 |  | 40 | 1438 | 1187 |
| Copepodites (C4→C5) | | 45 | 227 | 1880 |  | - | 125 | 1213 |
| *Pseudocalanus* spp./adults | | - | - | 84 |  | - | 13 | 67 |
|  | |  |  |  |  |  |  |  |

**Table S5.** Principal component analysis output for Figure 5.

| PC Eigenvalues %Variation Cum.%Variation |
| --- |
| 1 2.86 **47.7** 47.7 |
| 2 1.24 **20.7** 68.4 |
| 3 0.803 13.4 81.8 |
| 4 0.584 9.7 91.5 |
| 5 0.378 6.3 97.8 |

Vector scores

| Variable PC1 PC2 PC3 PC4 PC5 |
| --- |
| thr **-0.480** 0.264 -0.011 0.005 0.811 |
| val 0.104  **0.777** -0.193 0.546 -0.158 |
| leu **-0.521** -0.219 0.248 0.274 -0.048 |
| ile **-0.481** 0.127 0.492 0.108 -0.457 |
| phe **-0.388** 0.339 -0.380 -0.684 -0.293 |
| lys -0.325 **-0.383** -0.717 0.385 -0.145 |
|  |
| Sample scores in the Eastern Gotland Basin (EGB) |

| Samples | Score 1 | Score 2 | Samples | Score 1 | Score 2 |
| --- | --- | --- | --- | --- | --- |
| Phyto_1m_2015 | 3.73 | 6.7E-3 | EGBz-60-125m_2014 | 0.575 | -0.81 |
| Phyto 10m_2015 | 0.842 | -0.295 | EGBz-25-65m_2015 | -3.08 | 1.28 |
| Phyto 20m_2015 | 2.09 | 9.45E-2 | EGBz-25-65m_2015 | -2.29 | -1.69 |
| Phyto_30m_2015 | -1.01 | -0.661 | EGBz-0-25m_2015 | -0.796 | 1.15 |
| Phyto_1m_2015 | 3.16 | -0.16 | EGBz-0-25m_2015 | -1.32 | 1.13 |
| Phyto_10m_2015 | 3.91 | -0.739 | EGBz-0-25m_2015 | -0.762 | 1.01 |
| Phyto_20m_2015 | 3.61 | -2.46E-2 | EGBz-25-65m_2015 | -1.19 | 1.39 |
| Phyto_30m_2015 | 1.73 | 0.728 | EGBz-25-65m_2015 | -0.474 | 1.38 |
| Phyto_3.5m_2014 | -1.44 | -2.27 | EGBz-0-25m_2015 | -4.29E-2 | 0.587 |
| Phyto_12 m_2014 | 2.55E-2 | 0.154 | EGBz-0-25m_2015 | -0.239 | 0.989 |
| EGBz-0-10m_2014 | -1.09 | 0.141 | EGBz-25-65m_2015 | -0.7 | 0.9 |
| EGBz-0-10m_2014 | -0.414 | 0.305 | EGBz-0-25m_2015 | -0.231 | 1.4 |
| EGBz-0-10m_2014 | -0.694 | -1.85 | EGBz-0-25m_2015 | 0.965 | 0.832 |
| EGBz-10-60m_2014 | -0.349 | -1.3 | EGBz-0-25m_2015 | 1.76 | 0.942 |
| EGBz-10-60m_2014 | -0.459 | -1.77 | EGBz-0-25m_2015 | -2.94 | 1.36 |
| EGBz-10-60m_2014 | -7.19E-3 | -1.39 | EGBz-0-25m_2015 | 8.39E-2 | 1.12 |
| EGBz-10-60m_2014 | -1.01 | -1.6 | EGBz-25-65m_2015 | 0.446 | 0.752 |
| EGBz-60-125m_2014 | -1.05 | -1.15 |  |  |  |
| EGBz-60-125m_2014 | -1.44 | -1.15 |  |  |  |
| EGBz-60-125m_2014 | 0.106 | -0.799 |  |  |  |

**Table S5.** Principal component analysis output for Figure 6.

| PC Eigenvalues %Variation Cum.%Variation |
| --- |
| 1 3.27 54.4 54.4 |
| 2 1.19 19.8 74.2 |
| 3 0.642 10.7 84.9 |
| 4 0.465 7.7 92.7 |
| 5 0.312 5.2 97.9 |

Vector scores

| Variable PC1 PC2 PC3 PC4 PC5 |
| --- |
| thr **-0.422** 0.381 0.162 -0.529 0.528 |
| val 0.144  **0.840** 0.200 0.003 -0.455 |
| leu **-0.483** -0.169 0.465 -0.010 -0.001 |
| ile **-0.452** 0.111 0.216 0.756 0.017 |
| phe **-0.384** 0.245 -0.812 0.139 0.092 |
| lys **-0.465** -0.220 -0.108 -0.359 -0.711 |

Samples scores in the five Baltic Sea sub-basins (AB: Arkona Basin; BB: Bornholm Basin; SGB: southern Gotland Basin; EGB: eastern Gotland Basin)

| Samples | Score 1 | Score 2 | Samples | Score 1 | Score 2 |
| --- | --- | --- | --- | --- | --- |
| EGBz-0-10m_2014 | -1.6 | 0.374 | EGBz-0-25m_2015 | -0.377 | 1.11 |
| EGBz-0-10m_2014 | -0.791 | 0.589 | EGBz-0-25m_2015 | 0.926 | 0.484 |
| EGBz-0-10m_2014 | -1.17 | -2.06 | EGBz-0-25m_2015 | 1.86 | 0.242 |
| EGBz-10-60m_2014 | -0.742 | -1.59 | EGBz-0-25m_2015 | -3.24 | 1.49 |
| EGBz-10-60m_2014 | -0.912 | -2.05 | EGBz-0-25m_2015 | -1.13E-2 | 1.1 |
| EGBz-10-60m_2014 | -0.451 | -1.48 | EGBz-25-65m_2015 | 0.308 | 0.331 |
| EGBz-10-60m_2014 | -1.53 | -1.63 | WBz-0-25m_2015 | 0.993 | 5.42E-2 |
| EGBz-60-125m_2014 | -1.41 | -1.55 | WBz-0-25m_2015 | 2.24 | 0.142 |
| EGBz-60-125m_2014 | -1.77 | -1.69 | ABz-0-45m_2015 | 2.59 | -0.145 |
| EGBz-60-125m_2014 | -0.168 | -1.13 | ABz-0-45m_2015 | 2.79 | 0.225 |
| EGBz-60-125m_2014 | 0.395 | -1.33 | ABz-0-20m_2015 | 3.09 | -0.378 |
| EGBz-25-65m_2015 | -3.49 | 1.6 | ABz_0-20m_2015 | 0.604 | 0.258 |
| EGBz-25-65m_2015 | -2.87 | -1.81 | BBz_0-25m_2015 | 0.93 | -4.55E-2 |
| EGBz-0-25m_2015 | -1.02 | 1.13 | BBz_0-25m_2015 | 1.09 | 0.273 |
| EGBz-0-25m_2015 | -1.53 | 1.21 | SGB-0-20m_2015 | 2.86 | 0.336 |
| EGBz-0-25m_2015 | -1.09 | 1.09 | SGB-0-20m_2015 | 3.47 | -0.164 |
| EGBz-25-65m_2015 | -1.39 | 1.32 | WBz-0-15m_2015 | -0.233 | 0.543 |
| EGBz-25-65m_2015 | -0.584 | 1.34 | WBz-0-15m_2015 | 3.77 | -1.3E-2 |
| EGBz-0-25m_2015 | -0.233 | 0.282 |  |  |  |
| EGBz-0-25m_2015 | -0.408 | 0.835 |  |  |  |
| EGBz-25-65m_2015 | -0.905 | 0.73 |  |  |  |

**Figure S1.** Composition of individual amino acids (% of total amino acids) in two mesozooplankton size-fractions (100-300 µm and 300 µm) from three water bodies separated by the vertical water column stratification at the Eastern Gotland Basin in July 2014 and 2015. *Notes: the bottom water was collected only in 2014. Abbreviations: NEAAs – Non-essential amino acids; EAAs – Essential amino acids. The amino acids are indicated by their three-letter amino-acid code and explained in Materials and Methods.*

**Figure S2.** Delta values of δ^13^C between the three most informative essential amino acids (Ile, isoleucine; Leu, leucine; Lys, lysine, according to Larsen et al. (2009)) of POM samples (green circles, *n*=10) from this study and end-member values (bacteria (b); *n*=12) and microalgae (m); *n*=27)) from Larsen et al. (2013). POM samples are collected from the upper water column from 1m to max. 30m at the Eastern Gotland Basin (EGB) in July 2014 and 2015.
